# Minnelide suppresses GVHD and enhances survival while maintaining GVT responses

**DOI:** 10.1172/jci.insight.165936

**Published:** 2024-04-11

**Authors:** Sabrina N. Copsel, Vanessa T. Garrido, Henry Barreras, Cameron S. Bader, Brent Pfeiffer, Beatriz Mateo-Victoriano, Dietlinde Wolf, Miguel Gallardo, Sophie Paczesny, Krishna V. Komanduri, Cara L. Benjamin, Alejandro V. Villarino, Ashok K. Saluja, Robert B. Levy

**Affiliations:** 1 Department of Microbiology and Immunology,; 2 Department of Surgery, and; 3 Department of Pediatrics, University of Miami, Miller School of Medicine, Miami, Florida, USA.; 4 Sylvester Comprehensive Cancer Center,; 5 Department of Microbiology and Immunology, Medical University of South Carolina, Charleston, South Carolina, USA.; 6 Department of Medicine, and; 7 Department of Ophthalmology, University of Miami, Miller School of Medicine, Miami, Florida, USA.

**Keywords:** Immunology, Transplantation, Cellular immune response, Stem cell transplantation

## Abstract

Allogeneic hematopoietic stem cell transplantation (aHSCT) can cure patients with otherwise fatal leukemias and lymphomas. However, the benefits of aHSCT are limited by graft-versus-host disease (GVHD). Minnelide, a water-soluble analog of triptolide, has demonstrated potent antiinflammatory and antitumor activity in several preclinical models and has proven both safe and efficacious in clinical trials for advanced gastrointestinal malignancies. Here, we tested the effectiveness of Minnelide in preventing acute GVHD as compared with posttransplant cyclophosphamide (PTCy). Strikingly, we found Minnelide improved survival, weight loss, and clinical scores in an MHC-mismatched model of aHSCT. These benefits were also apparent in minor MHC–matched aHSCT and xenogeneic HSCT models. Minnelide was comparable to PTCy in terms of survival, GVHD clinical score, and colonic length. Notably, in addition to decreased donor T cell infiltration early after aHSCT, several regulatory cell populations, including Tregs, ILC2s, and myeloid-derived stem cells in the colon were increased, which together may account for Minnelide’s GVHD suppression after aHSCT. Importantly, Minnelide’s GVHD prevention was accompanied by preservation of graft-versus-tumor activity. As Minnelide possesses anti–acute myeloid leukemia (anti-AML) activity and is being applied in clinical trials, together with the present findings, we conclude that this compound might provide a new approach for patients with AML undergoing aHSCT.

## Abstract 2

Water-soluble triptolide analog minnelide augments overall survival and diminishes GVHD severity following allogeneic and xenogeneic HSCT while preserving GVT activity.

## Graphical abstract

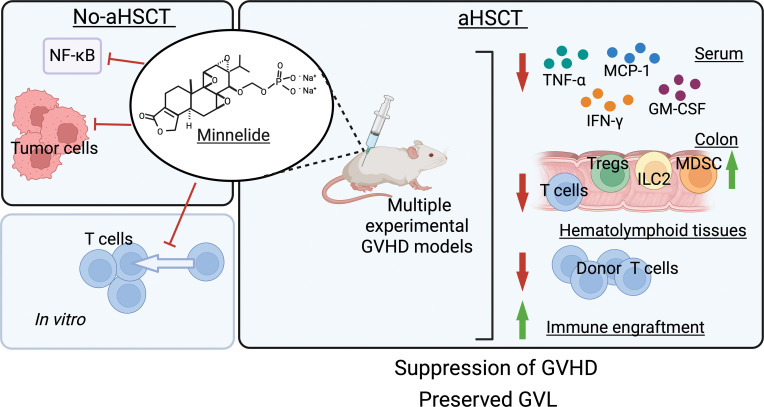

## Introduction

Allogeneic hematopoietic stem cell transplantation (aHSCT) is the preferred curative therapy for high-risk and/or relapsed hematological malignancies because of its desirable anti-leukemia/anti-lymphoma (graft-versus-tumor, GVT) effect (1–3). Although GVT effects are mainly mediated by donor-derived alloreactive T cells, this population is also responsible for the development of graft-versus-host disease (GVHD) (4–6). Despite current prophylactic strategies, GVHD is a major cause of morbidity and mortality following aHSCT, and patients refractory to the first-line standard regimens for GVHD treatment have a poor prognosis. Although the use of posttransplant cyclophosphamide (PTCy) has provided an advance in treatment for patients with aHSCT (7–12), new strategies are still needed to improve the complex balance between immune reconstitution and immunosuppression while preserving the beneficial GVT effect.

Triptolide is the most potent bioactive compound isolated from the traditional Chinese herb *Tripterygium wilfordii* Hook F, which exhibits immunosuppressive, antiinflammatory, and antitumor activities (13–18). Triptolide holds strong promise as an immunosuppressing agent based on several studies that examined its potential therapeutic effect for GVHD prophylaxis and maintenance of GVT activity in transplant recipients (19–27). Therapeutic use of triptolide and many of its derivatives is limited by issues with solubility, a narrow therapeutic window, and toxicity (28). However, Minnelide (14-*O*-phosphonooxymethyltriptolide disodium salt) is a highly water-soluble prodrug of triptolide converted to its active form by phosphatases present throughout the body, including the blood stream (29). Minnelide also has a more favorable toxicity profile than triptolide and is now being tested in preclinical models of multiple solid tumors (29–33) and acute myeloid leukemia (AML) (34). In addition, Minnelide is being pursued in a phase I clinical trial for patients with AML.

Herein, we present preclinical assessment of Minnelide as a prophylactic therapy for GVHD in multiple murine and humanized models of aHSCT. In a complete MHC-mismatched model, we show that Minnelide effectively prevented GVHD and markedly increased overall survival of recipients with daily treatment of a short regimen of 2 weeks from day of transplant. Notably, together with decreased donor T cell infiltration early after aHSCT, several adaptive and innate regulatory cell populations were increased following Minnelide treatment. These included regulatory T cells (Tregs), type 2 innate lymphoid cells (ILC2s), and myeloid-derived stem cells (MDSCs) in several target tissues, including the spleen, colon, and lung. Treatment also promoted hematopoietic engraftment and importantly, GVT responses were effectively maintained. Notably, Minnelide could directly inhibit T cell proliferation and reduced Th1 and CD8^+^ T cell IFN-γ production early after transplantation. The use of NF-κB reporter mice demonstrated that a brief 2-day treatment with Minnelide reduced activation of the NF-κB pathway, consistent with the above findings. Importantly, to directly assess its ability to regulate human T cell–mediated GVHD, NSG mice transplanted with mobilized human peripheral blood mononuclear cells (HuPBMCs) were treated with Minnelide, which ablated in vivo human T cell proliferation and prevented xenogeneic GVHD (xGVHD). Additionally, triptolide suppressed human CD4^+^ and CD8^+^ T cell proliferation in response to alloantigen stimulation in vitro. Therefore, direct inhibition of T cells together with an immunoregulatory cell network may account for Minnelide’s suppression of GVHD. In total, these findings support the notion that Minnelide may provide new opportunities for developing translational strategies for the prevention of GVHD while maintaining GVT and potentially providing direct antitumor effects in some tumor models.

## Results

### Minnelide prevents GVHD and promotes hematopoietic engraftment following aHSCT.

**Figure 1:**
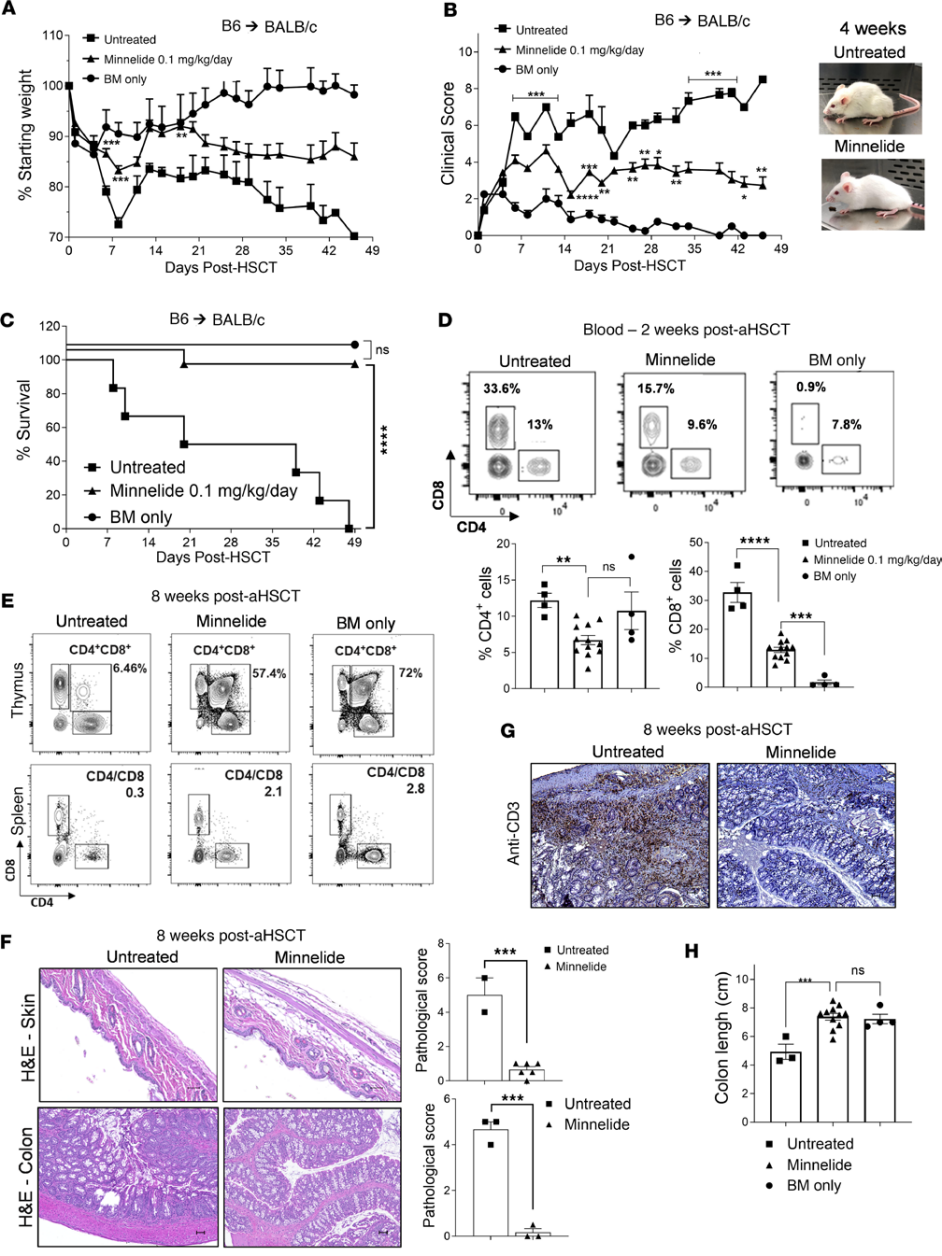
Recipients treated with Minnelide exhibited diminished acute GVHD after MHC-mismatched aHSCT. (**A**–**C**) An aHSCT was performed utilizing a B6→BALB/c donor and recipient mouse model (day –1: 7.5–8.5 Gy; day 0: 5.5 × 10^6^ TCD BM cells and pooled splenocytes containing 0.8 × 10^6^ T cells) and recipients were treated with 0.1 mg/kg Minnelide from day −2 to 28 after transplantation. Weight loss (**A**), clinical scores (**B**), and GVHD survival (**C**) are presented (*n* = 8 untreated, *n* = 16 Minnelide-treated, and *n* = 4 BM-only mice). (**D**) Representative flow cytometry contour plots and frequency of CD4^+^ and CD8^+^ cells in the blood on day 14 after aHSCT. (**E**) Eight weeks after aHSCT, thymic and splenic T cell populations were evaluated and representative flow cytometry plots are shown. (**F**) Representative H&E staining and pathology scores (on the right) (*n* = 2–6) from the skin and colon 8 weeks after aHSCT. Original magnification, ×200 (top) and ×100 (bottom), respectively. (**G**) Representative photographs of colon anti-CD3 staining 8 weeks after transplantation. Original magnification, ×100. (**H**) Increased colon length in recipients of Minnelide treatment 8 weeks after aHSCT. These data are representative of 4 independent aHSCT experiments in this model. Clinical scores were compared using Benjamini-Krieger-Yekutieli–corrected multiple *t* tests over time. Groups were compared using 1-way ANOVA with Tukey’s multiple-comparison test and a log-rank test was used for survival analyses. *P < 0.05; **P < 0.01; ***P < 0.001; ****P < 0.0001. Data are presented as mean ± SEM.

To determine whether the triptolide prodrug Minnelide affects the immune compartment, in the absence of an inflammatory stimulus, naive BALB/c mice were treated daily for 30 days with either 0.1 or 0.2 mg/kg of this compound. No changes in the overall splenic and lymph node CD4^+^ and CD8^+^ T cell, Treg, and B cell compartments were detected (Supplemental Figure 1; supplemental material available online with this article; undefinedDS1). Transplants were then performed to assess the capacity of Minnelide treatment to ameliorate GVHD. Strikingly, we found that daily doses of 0.1 mg/kg Minnelide from day –1 to day 28 improved weight loss, GVHD clinical score, and overall survival as compared with the untreated group in a complete MHC-mismatched (B6→BALB/c) aHSCT model of GVHD (Figure 1, A–C). Animals receiving bone marrow (BM) only and treated with Minnelide exhibited no differences in weight loss, clinical score, lethality, or change in T cell frequency, indicating GVHD in untreated controls resulted primarily from allogeneic donor T cells (Supplemental Figure 2, A–D). Given the short half-life of Minnelide, we also tested a dose of 0.05 mg/kg twice daily and obtained similar results (Supplemental Figure 3, A–C). Ultimately, a dose of 0.1 mg/kg once per day was selected for all subsequent experimental treatments. Blood monitoring at 2 weeks after transplantation showed reduced donor CD4^+^ and CD8^+^ T cells in Minnelide-treated versus nontreated animals (Figure 1D). At 2 months after transplantation, CD4^+^ and CD8^+^ T cell analyses demonstrated improved frequency and ratios of these T cell subsets in the spleen, similar to the BM-only group (Figure 1E and Supplemental Figure 3D). We also noted thymocyte numbers were elevated and contained a normal number and percentage of CD4^+^CD8^+^ double-positive (DP) thymocytes (Figure 1E and Supplemental Figure 3E). Histological evaluation of Minnelide-treated mice indicated a significant decrease in skin involvement, as assessed by overall thickening and fibrosis 2 months after aHSCT (Figure 1F). Colons from untreated mice exhibited mucosal thickening and severe inflammation with villi distortion. However, colons from Minnelide-treated animals showed no disruption of villi architecture, mild inflammation, a marked reduction in infiltrating CD3^+^ T cells, and improved length (Figure 1, F–H). Importantly, all effects on colon pathophysiology were visible as early as 1 week after transplantation (Supplemental Figure 3F).

**Figure 2:**
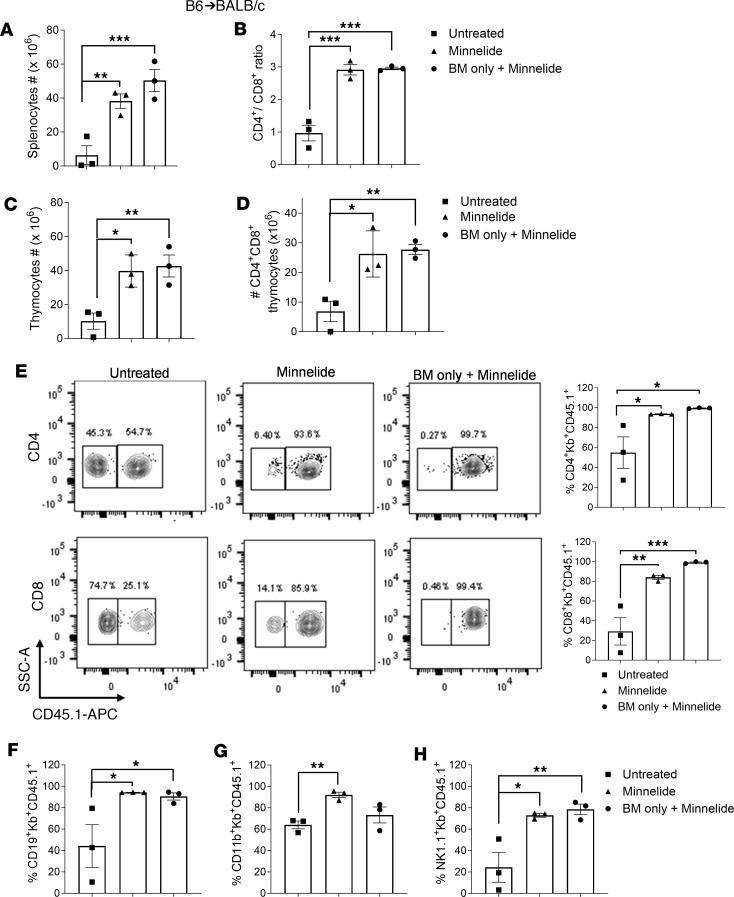
Minnelide treatment promotes lymphoid engraftment after MHC-mismatched aHSCT. (**A**–**H**) An aHSCT was performed utilizing the same model as in Figure 1 (B6→BALB/c donor and recipient strain combination), but transplanted BM was derived from congenic B6-CD45.1 donors and T cells from B6-CD45.2 donors. Recipients were treated with 0.1 mg/kg Minnelide from day −2 to 28 after transplantation. Seven weeks after aHSCT, splenic and thymic tissues were analyzed for total cell numbers (**A** and **C**), splenic CD4^+^/CD8^+^ ratio (**B**), and CD4^+^CD8^+^ DP thymocytes (**D**). (**E**) Representative flow cytometry contour plots and frequency of donor (transplanted hematopoietic progenitors) BM–derived (Kb^+^CD45.1^+^) CD4^+^ and CD8^+^ cells in the spleen seven weeks after aHSCT are shown. Kb, MHC H2K^b^. Frequency of splenic CD19^+^ (**F**), CD11b^+^ (**G**), and NK1.1^+^ (**H**) cells derived from donor BM 7 weeks after transplantation. GVHD, *n* = 3; Minnelide, *n* = 3; BM only + Minnelide, *n* = 3. Groups compared using 1-way ANOVA with Dunnett’s multiple-comparison test. *P < 0.05; **P < 0.01; ***P < 0.001. Data are presented as mean ± SEM.

An independent experiment was performed using B6 congenic donor BM (CD45.1) and T cells (CD45.2) to assess post-HSCT multiple-lineage engraftment. Minnelide treatment augmented splenocyte and thymocyte (as noted above) numbers, including higher CD4^+^/CD8^+^ splenic ratios and DP thymocyte numbers 7 weeks after aHSCT (Figure 2, A–D). Immune lineages involved in the pathogenesis of GVHD were then assessed by flow cytometry (CD45.1, MHC H2K^b^), including T cells, NK cells, and antigen-presenting cells (APCs). Mice receiving Minnelide treatment exhibited CD4^+^ and CD8^+^ T cell engraftment from donor BM, reaching greater than 85% at 7 weeks after transplantation, which was indistinguishable from BM-only transplanted mice (Figure 2E). Regarding B cells (CD19^+^), similar results were obtained with overall engraftment and again was comparable to BM alone (Figure 2F). Additionally, CD11b- and NK1.1-expressing populations indicated predominant repopulation from donor BM–derived progenitors and the levels were comparable to BM-alone transplanted recipients (Figure 2, G and H).

Next, we compared Minnelide to PTCy treatment, a novel and widely used prophylactic GVHD treatment. Mismatched aHSCT recipients were administered Cy (50 mg/kg) on days 3 and 4 after aHSCT (35, 36) or Minnelide as described above. Notably, the treatments were virtually identical in preventing GVHD and significantly superior compared with untreated mice, as assessed by survival, GVHD clinical score, colon length, splenic CD4^+^ and CD8^+^ naive/effector cells, and thymic T cells (Supplemental Figure 4, A–D). Additionally, Minnelide was administered over different time periods: twice (days 3 and 4 to mimic PTCy), 1 week (days 1–7), and 2 weeks. Day 3 and 4 treatment did have some beneficial effect on mouse survival. Administration for the first week resulted in improved survival and some transient clinical score improvement versus days 3 and 4 (Supplemental Figure 4, E and F). Notably, administration for 14 days resulted in 100% survival and diminished GVHD scores (Supplemental Figure 4, E and F) that correlated with longer GI length (data not shown).

In a model of MHC-matched, minor antigen–disparate donors/recipients (C3H.SW→B6), daily Minnelide treatment also reduced GVHD clinical scores and augmented the numbers of lymph node cells in recipients (Supplemental Figure 5A). However, in a chronic GVHD model (B10.D2→BALB/c), no effect on clinical scores was observed following Minnelide therapeutic administration in the second month after aHSCT (Supplemental Figure 5B). In total, these findings demonstrated that Minnelide treatment prevented experimental GVHD in independent models and promoted multiple-lineage chimerism from transplanted donor cells.

### Minnelide limits donor T cell proliferation and proinflammatory cytokine production during acute GVHD.

**Figure 3:**
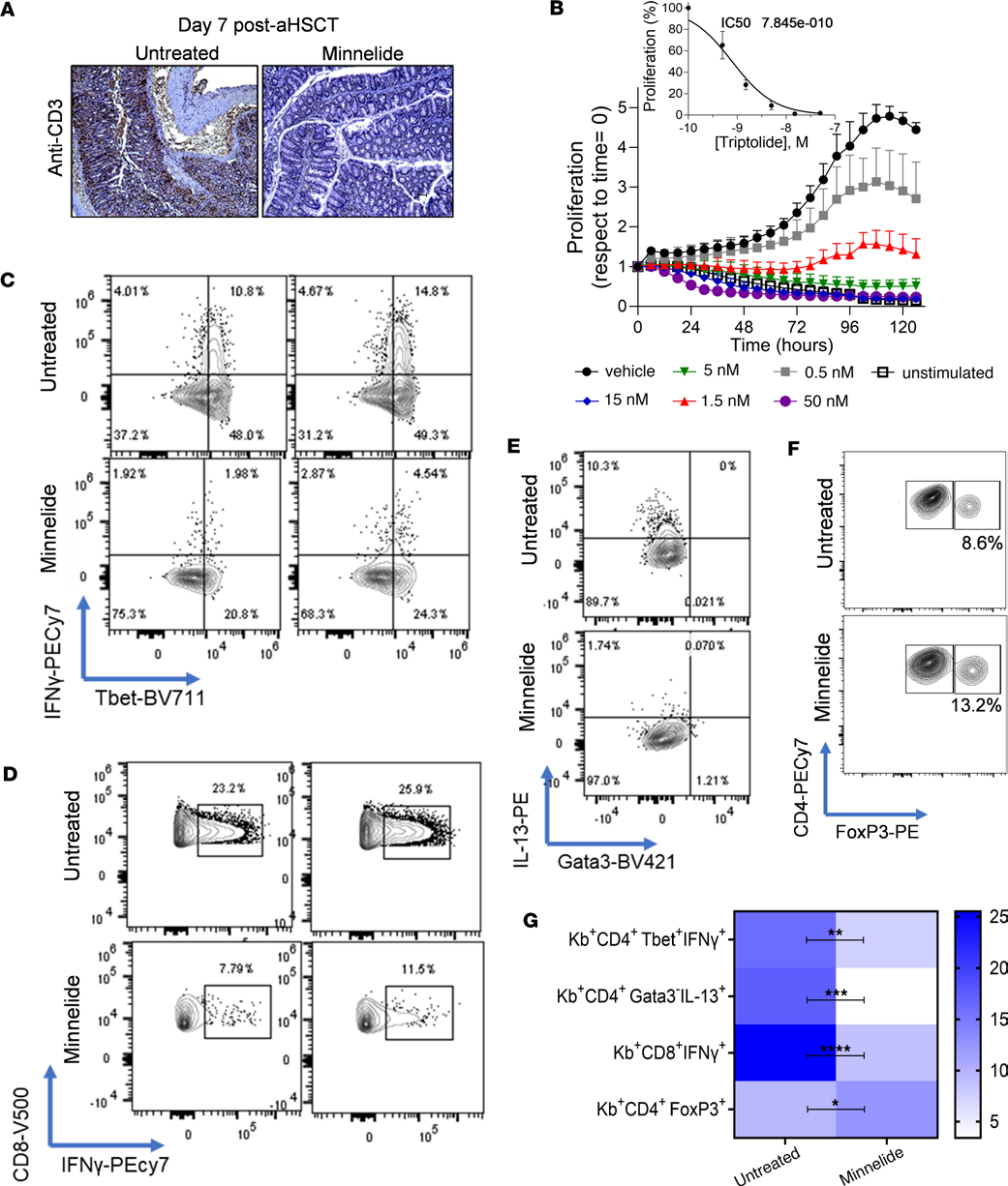
Minnelide can inhibit T cell proliferation and decrease T cell proinflammatory cytokines early after MHC-mismatched aHSCT. Recipients were treated with 0.1 mg/kg Minnelide from day −2 to 7 after transplantation, and on day 7 colons and spleens were evaluated. (**A**) Representative photographs of colon anti-CD3 staining show a marked decrease in CD3^+^ cell infiltrate. Original magnification, ×100. (**B**) Purified T cells were seeded in 96-well plates and treated with different doses of triptolide for 120 hours. A dose-dependent decrease in proliferation was observed in response to 0.5–50 nM triptolide. A significant and lasting reduction in proliferation was observed in response to treatment. (**C**–**G**) On day 7 after aHSCT, spleens were analyzed for frequency of donor (**C**) Th1 cells producing IFN-γ, (**D**) CD8^+^ cells producing IFN-γ, (**E**) Th22 cells producing IL-13, and (**F**) CD4^+^FoxP3^+^ Tregs. (**G**) Summary data of the frequency of the indicated donor (MHC H2K^b^–positive, Kb^+^) cell populations. These data were pooled from 2 independent aHSCT experiments (untreated, *n* = 5; Minnelide, *n* = 5). Groups were compared using a 2-tailed, unpaired *t* test. *P < 0.05, **P < 0.01, ***P < 0.001, ****P < 0.0001. Data are presented as mean ± SEM.

Cellular events within the first 1–2 weeks after transplantation are critical to the development of GVHD. Notably, less CD3^+^ cell infiltrate in Minnelide versus untreated mice was detected at early time points (1 week after aHSCT) (Figure 3A). To extend this finding and further investigate the effect by Minnelide on cellular events and signaling pathways involved in early T cell responses, we evaluated T cell proliferation in response to Minnelide treatment. Minnelide requires phosphatase activity that leads to activation and passage through cell membranes. Therefore, to examine T cell proliferation in vitro, it was necessary to employ triptolide and we identified sharply reduced splenic T cell proliferation in a time- and concentration-dependent manner (Figure 3B). In vivo, following aHSCT, recipients were administered 0.1 mg/kg Minnelide for up to 1 week (these animals showed reduced GVHD clinical scores as anticipated; data not shown) and the splenic T cell response was evaluated. After 6 hours of in vitro stimulation with an anti-CD3 mAb, splenocytes showed a marked reduction in CD4^+^ (Th1) and CD8^+^ T cells producing IFN-γ (Figure 3, C and D) and Th22 cells producing IL-13 (Figure 3E). While Minnelide did not alter Treg frequency and subsets under homeostatic conditions (Supplemental Figure 1D and data not shown), during the first week after aHSCT in Minnelide-treated animals, the frequency of CD4^+^FoxP3^+^ Treg cells was found to be elevated (Figure 3F) and a summary of 2 independent experiments illustrates these proinflammatory T and Treg cell observations (Figure 3G). Perhaps the Treg increase could result from lymphopenic conditions due to Minnelide’s reduction of GVHD, by decreasing T cell expansion early after aHSCT.

**Figure 4:**
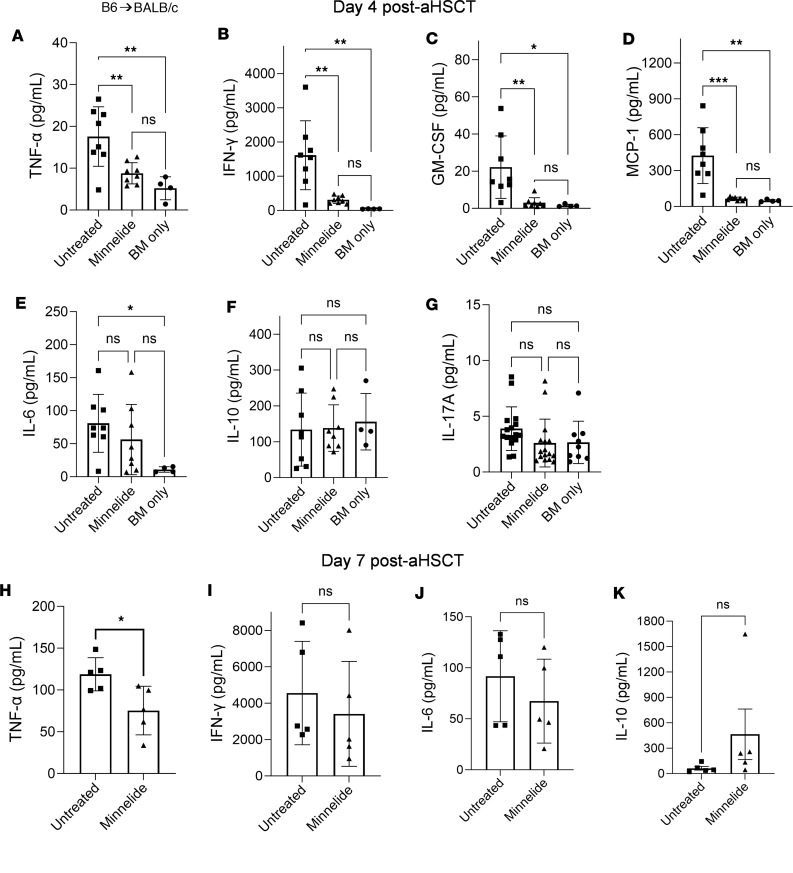
Minnelide decreases cytokine storm in recipient mice following MHC-mismatched aHSCT. (**A**–**K**) Using the major BM transplantation model described in Figure 1, recipients were treated with 0.1 mg/kg Minnelide from day −2 to 7 after transplantation, and on days 4 (**A**–**G**) and 7 (**H**–**K**) serum was collected via cardiac puncture for cytokine quantification using the LEGENDplex Mouse Inflammation Panel (see Supplemental Methods). The specific cytokines evaluated are indicated on the *y* axis of each graph. Groups were compared using 1-way ANOVA with Tukey’s multiple-comparison test. *P < 0.05; **P < 0.01; ***P < 0.001. Data are presented as mean ± SEM.

Next, to examine the effect of Minnelide on proinflammatory cytokine production early after aHSCT, serum was collected from recipients during the first week after MHC-mismatched transplantation. Significant decreases were identified in several inflammatory cytokines on day 4, specifically TNF-α, IFN-γ, MCP-1, and GM-CSF (Figure 4, A–G). Only TNF-α remained decreased on day 7 (Figure 4, H–K). NF-κB is a key signaling pathway involved that induces inflammatory cytokine production in immune cells. Because Minnelide has been shown to suppress NF-κB signaling in vitro (37), we next determined whether it could also do so in vivo, under inflammatory conditions. Since bacterial products (e.g., LPS) leak across the gut wall following conditioning and transplantation, NF-κB reporter mice (FVB NGL, see Methods) were injected with 1 mg/kg LPS with or without 0.1 mg/kg Minnelide and 4 hours later luciferase was measured as an indicator of NF-κB activity. Strikingly, we found a substantial reduction in NF-κB activation in Minnelide-treated mice relative to controls (Supplemental Figure 6).

### Minnelide treatment increases innate and adaptive regulatory cell populations that promote immunosuppressive activity in acute GVHD target tissues following MHC-mismatched aHSCT.

**Figure 5:**
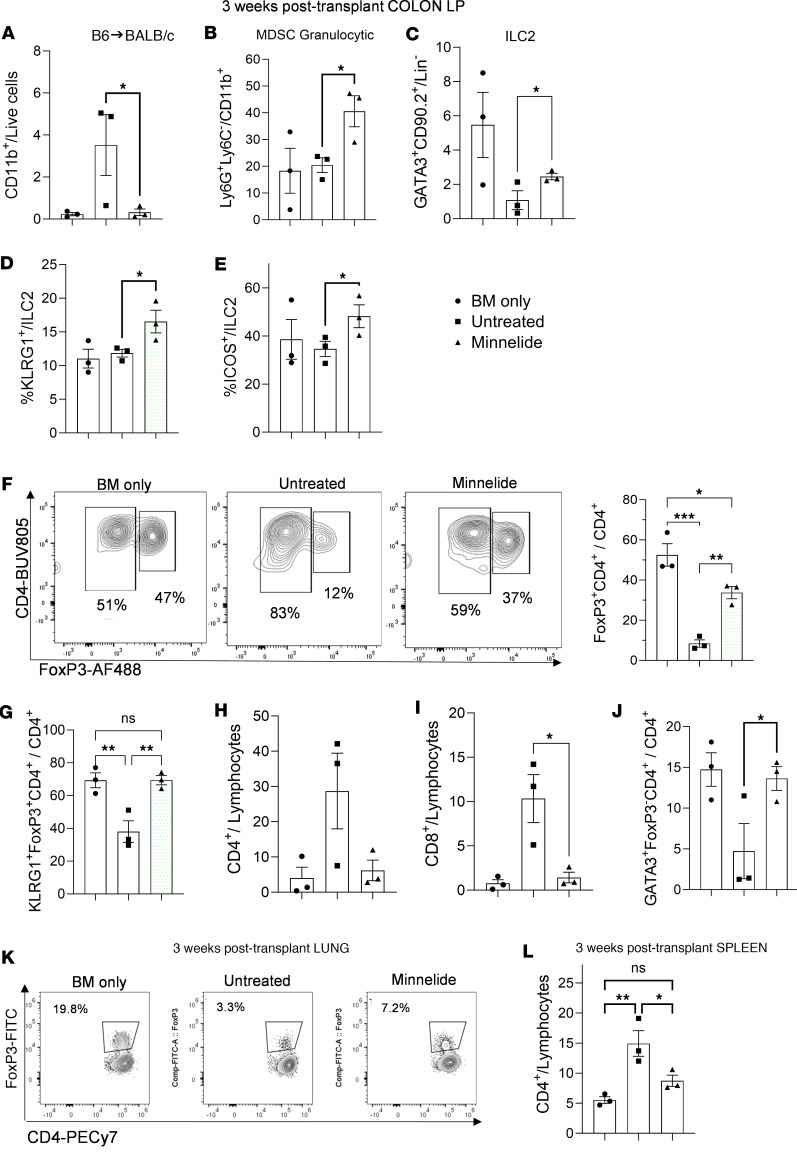
Minnelide treatment increases innate and adaptive regulatory cell populations that promote immunosuppressive activity in GVHD target tissues following MHC-mismatched aHSCT. Using the major BM transplantation model, B6→BALB/c (described in Figure 1), recipients were treated with 0.1 mg/kg Minnelide from day −2 to day +20 after transplantation. On day 20 after aHSCT, lamina propria (LP) from colon (**A**–**J**) as well as lung lymphocytes (**K**) was evaluated. Data are presented as frequency of colonic CD11b^+^ cells (**A**), frequency of MDSCs (Ly6G^+^Ly6C^–^CD11b^+^) (**B**), frequency of ILC2s (GATA3^+^CD90.2^+^Lin^–^) (**C**), frequency of KLRG1^+^cells within the ILC2 population (**D**), and ICOS^+^ cells (**E**). (**F**) Representative flow cytometry contour plots and frequency of Tregs (CD4^+^FoxP3^+^) in colonic LP from BM-only, untreated (received BM + T cells), and Minnelide-treated (received BM + T cells) mice (*n* = 3). Frequency of LP T cell subsets, CD4^+^FoxP3^+^KLRG1^+^ (**G**), total CD4^+^ (**H**), total CD8^+^ (**I**), and Th2 (CD4^+^FoxP3^–^GATA3^+^) (**J**). (**K**) Representative flow cytometric contour plots of Treg cells in the lung (CD4^+^FoxP3^+^CD4^+^). (**L**) Frequency of CD4^+^ and CD8^+^ cells in the spleen 3 weeks after aHSCT. Groups were compared using 1-way ANOVA with Tukey’s multiple-comparison test. *P < 0.05; **P < 0.01; ***P < 0.001.

An independent experiment was performed using a complete MHC-mismatched (B6→BALB/c) aHSCT model of GVHD. As shown in Figure 1, daily doses of 0.1 mg/kg Minnelide improved weight loss and GVHD clinical score as compared with the untreated group (Supplemental Figure 5C). Three weeks after transplantation, colonic lamina propria from each group of mice were analyzed and we found a reduced frequency of CD11b^+^ cells together with increased levels of regulatory populations, including MDSCs and ILC2s (Figure 5, A–C) that expressed substantial levels of KLRG1 and ICOS (Figure 5, D and E).

Notably, Tregs were found to be significantly increased in the colon in 2 independent experiments in Minnelide-treated animals compared with untreated recipients (Figure 5F). Moreover, a terminal differentiation marker, KLRG1, was also found to be higher in this regulatory population in mice treated with Minnelide (Figure 5G). Furthermore, CD4^+^ Th2 (Gata3^+^) frequency was elevated together with decreased CD4^+^ conventional T (Tconv) and CD8^+^ T cells in the colon 3 weeks after transplantation (Figure 5, H–J). We also examined another frequent GVHD target tissue, i.e., the lung, and found Treg levels were doubled in this compartment as well approximately 3 weeks after aHSCT (Figure 5K). These increases contrast with the overall decrease in the frequency of T cells in the colon at this time point (Figure 3A) as well as in the spleen (Figure 5L). Moreover, CD11c-expressing cells in the GI tract were examined at several time points (1 week, not shown; 8 weeks, see Supplemental Figure 5D) and the levels of CD11c^+^ cells were reduced in the Minnelide-treated mice (Supplemental Figure 5D). These new data sets provide insight into regulatory pathways that may underlie immune mechanism(s) induced by Minnelide treatment.

### Minnelide treatment in recipient mice preserves functional immunity following aHSCT, as assessed by effective GVT and skin allograft rejection responses.

**Figure 6:**
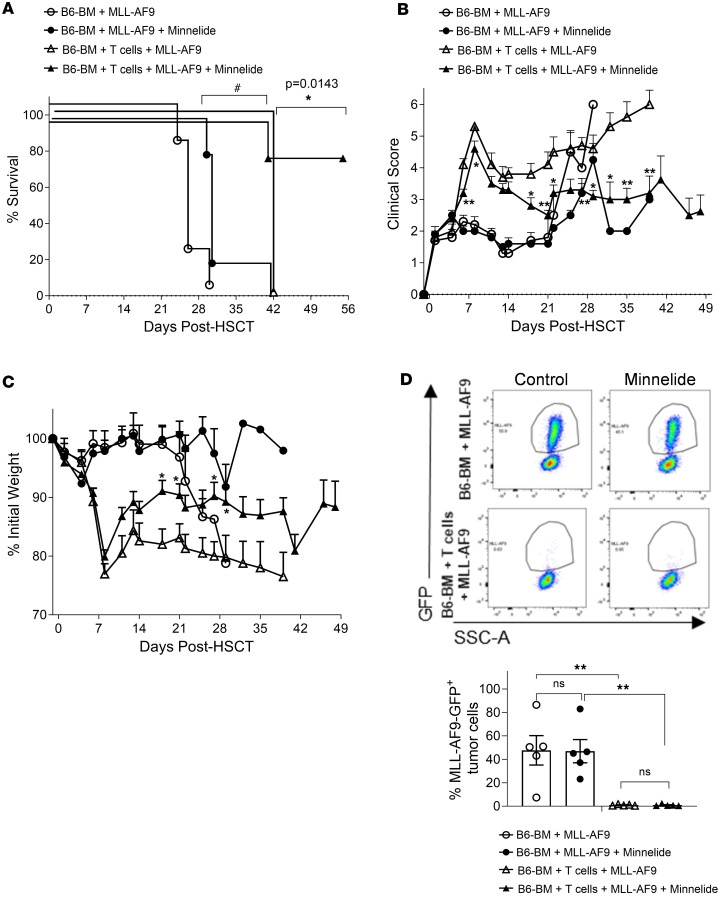
Recipients treated with Minnelide exhibited maintained GVT, while GVHD was ameliorated after MHC-mismatched aHSCT. (**A**–**C**) aHSCT was performed utilizing a B6→BALB/c donor and recipient mouse model and recipients were treated with 0.1 mg/kg Minnelide from day −2 to 28 after transplantation. GVHD survival (**A**), clinical scores (**B**), and weight loss (**C**) are presented (*n* = 8 GVHD, *n* = 16 Minnelide, and *n* = 4 BM-only mice). (**D**) Tumor burden in recipient blood on day 22 after BM transplantation. The data are from 2 independent experiments. Clinical scores were compared using Benjamini-Krieger-Yekutieli–corrected multiple *t* tests over time. Groups were compared using 1-way ANOVA with Tukey’s multiple-comparison test or log-rank test for survival analyses. *P < 0.05 for B6 BM + T cells + MLL-AF9 vs. B6 BM + T cells + MLL-AF9 + Minnelide; ^#^*P* < 0.05 for B6 BM + MLL-AF9 vs. B6 BM + MLL-AF9 + Minnelide; **P < 0.01. Data are presented as mean ± SEM.

To achieve relapse-free survival, amelioration of GVHD must be accompanied by maintenance of potent GVT responses. Here, we report that Minnelide reduces donor T cells and cytokine production in the context of GVHD. However, there is also evidence that Minnelide has anti–tumor cell activity. Therefore, several established mouse tumors used for GVT studies were examined to determine their susceptibility to direct killing by this triptolide prodrug. First, we tested the effect of triptolide on P815 mastocytoma and A20 B cell lymphoma (H2^d^) tumor cell lines in vitro. Triptolide exhibited potent dose-dependent killing activity against both tumor cell lines, beginning at low doses (P815 and A20 IC_50_: 1.942 × 10^–9^ M and 1.600 × 10^–9^ M, respectively) (Supplemental Figure 7, A and B). Consistent with the in vitro findings, nontransplanted mice injected with 2 × 10^6^ A20 lymphoma cells showed significant tumor growth ablation in mice treated with Minnelide at a GVHD-regulating dose (0.1 mg/kg/day) (Supplemental Figure 7C). In contrast, Minnelide did not prevent tumor progression in untransplanted BALB/c mice injected with AF9^GFP^ (H2^d^) cells and therefore could provide a useful preclinical model for a GVT response independent of Minnelide. Next, we assessed whether Minnelide treatment preserved GVT responses in a clinically relevant oncofusion protein–induced leukemia. MLL-AF9^GFP^ cells were administered into BALB/c mice together with donor B6 T and BM cells 1 day after irradiation (8 Gy). Mice were treated with 0.1 mg/kg Minnelide for 30 days. As anticipated, Minnelide treatment reduced GVHD severity, regardless of whether animals were injected with MLL-AF9 leukemia cells (Figure 6, A–C). Importantly, T cell–mediated GVT activity was preserved, as assessed by absence of MLL-AF9^GFP^ tumor cells in peripheral blood 3 weeks after aHSCT (Figure 6D). Furthermore, when blood, peripheral lymph nodes, spleen, and BM were examined 6 weeks after transplantation, we noted that antitumor activity in Minnelide-treated recipients was as effective as in recipients that were not treated with this drug (Supplemental Figure 8).

**Figure 7:**
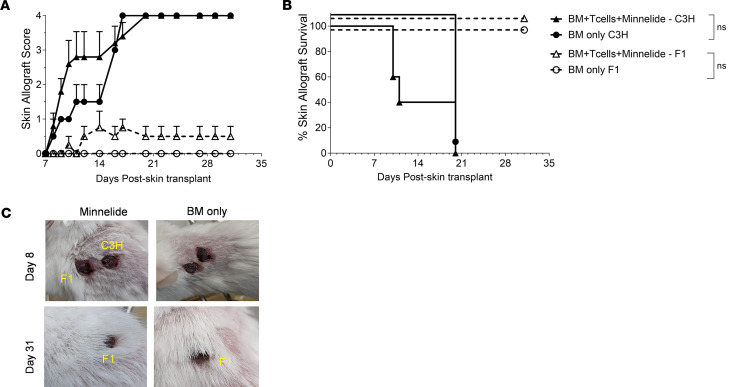
Functional immunity is intact in Minnelide-treated recipients. Two months after aHSCT, recipients received 2 skin grafts, applied on the trunk of each mouse, 1 from B6 × BALB/c F_1_ (H2^b/d^) mice and 1 from C3H/HeJ third-party (H2^k^) donors. Grafts were assessed and scored on the indicated days (Minnelide *n*= 5; BM only *n* = 2). (**A**) Allograft score. Graft scoring was performed as follows: 0, intact graft and healthy appearance; 1, inflamed graft, but without signs of necrosis observed; 2, inflamed graft and less than 25% necrosis observed; 3, inflamed graft and between 25% and 75% necrosis observed; and 4, greater than 75% necrosis detected or loss of graft. (**B**) Allograft survival. All mice accepted the F_1_ (B6 × BALB/c) skin grafts, whereas all C3H/HeJ grafts were rejected in both Minnelide and BM-only transplant recipients by day 21. (**C**) Representative photographs of skin grafts present on recipient mice on days 8 and 31 from both groups. Groups were compared using log-rank for survival analyses. Data are presented as mean ± SEM.

Next, we investigated the impact of Minnelide on tolerance to donor and recipient antigens, placing multiple skin grafts onto individual aHSCT recipient mice. Two heterotopic skin grafts, specifically one from B6 × BALB/c F_1_ (H2^b/d^) mice, and one from C3H/HeJ “third-party” (H2^k^) donors were applied to the trunk of each BALB/c mouse 2 months after aHSCT (by this time after aHSCT, 100% mortality had occurred in untreated GVHD animals). Strikingly, Minnelide-treated animals transplanted with donor T cells plus BM accepted the F_1_ skin grafts, as did recipients transplanted with BM only. These results demonstrated tolerance to donor and host alloantigens in both groups. In contrast, these same recipient groups rejected complete MHC-mismatched third-party C3H/HeJ allografts by 3 weeks after skin grafting (Figure 7, A–C). These findings together with the GVT data above provide evidence of functional immunity in recipients of BM plus T cells treated with Minnelide. Moreover, such immunity was accompanied by the establishment of tolerance to donor and host but not “third-party” alloantigens.

### Minnelide diminishes human donor T cell expansion and abrogates xGVHD.

**Figure 8:**
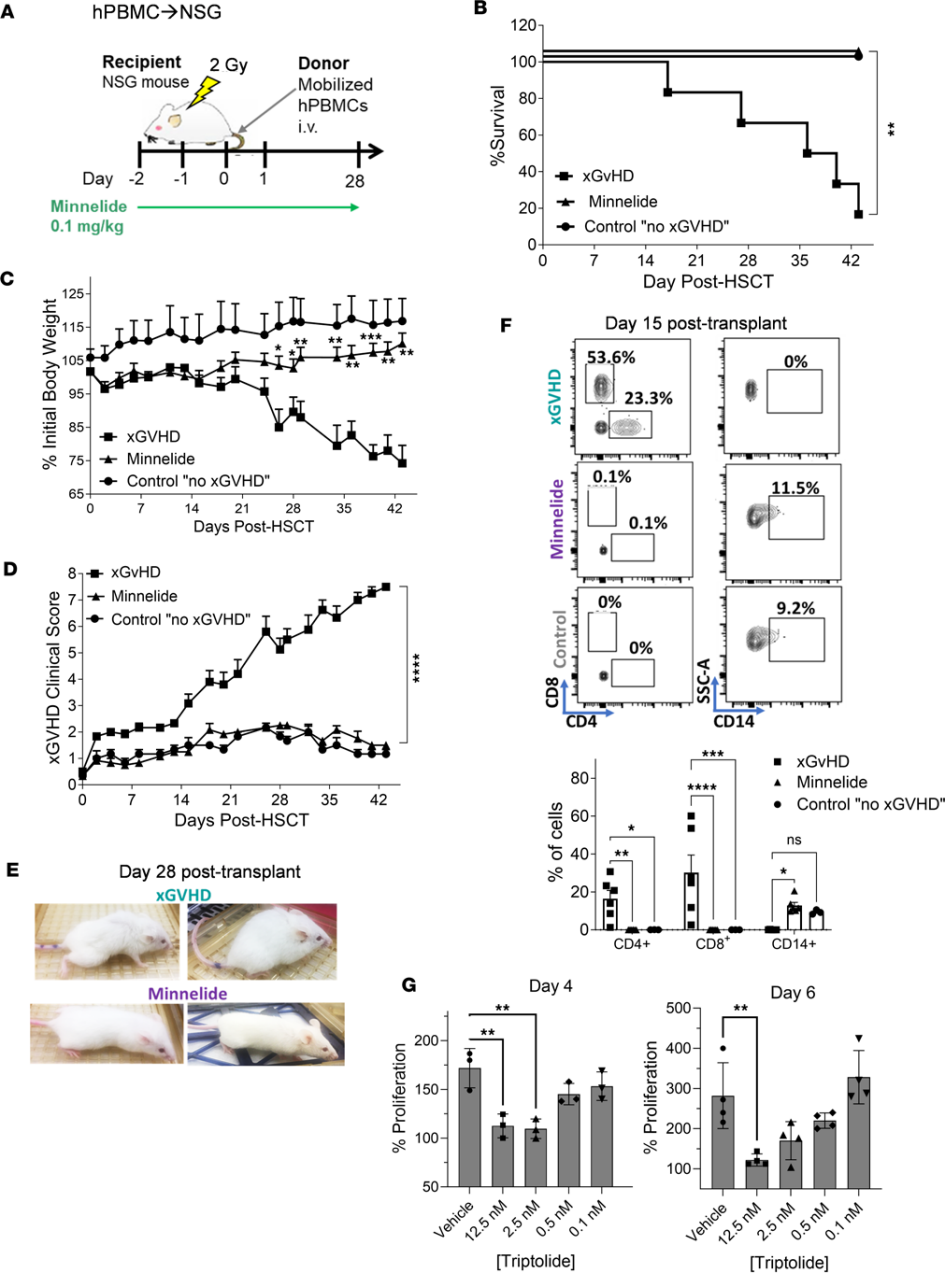
Treatment with Minnelide reduced xGVHD. (**A**–**F**) NSG recipient mice were irradiated (2 Gy) and the following day underwent transplantation with human mobilized PBMCs (6 × 10^6^). Recipients were treated with 0.1 mg/kg Minnelide from day −2 to 28 after transplantation. Schematic of experimental outline (**A**), overall survival (**B**), weight loss (**C**), and xGVHD clinical scores (**D**) are presented (*n* = 6 xGVHD or Minnelide and *n* = 3 control [no xGVHD] mice). (**E**) Representative photographs of xGVHD and Minnelide-treated recipients 4 weeks after transplantation. (**F**) Flow cytometry contour plots and frequency of human CD4^+^, CD8^+^, and CD14^+^ cells in the blood 2 weeks after transplantation. These data are representative of 2 independent (*n* = 6/group) human-to-mouse HSCTs. Clinical scores were compared using Benjamini-Krieger-Yekutieli–corrected multiple *t* tests over time. (**G**) Proliferation assays of CellTrace Violet–labeled human PBMCs stimulated by either anti-CD3 (OKT3 mAb) in vitro for 4 days and a 1-way mixed lymphocyte reaction for 6 days. Groups compared using 2-way ANOVA with Dunnett’s multiple-comparison test or log-rank for survival analyses. *P < 0.05; **P < 0.01; **P < 0.001; ****P < 0.0001. Data are presented as mean ± SEM.

To assess whether Minnelide regulates human T cell responses, we used an xGVHD model involving HuPBMCs from mobilized donors (Supplemental Figure 9). Recipient NSG mice were irradiated (2 Gy) on day –1 and the following day received 6 × 10^6^ mobilized HuPBMCs. Groups were either untreated (controls) or treated with 0.1 mg/kg Minnelide for 30 days (Figure 8A). Mobilized human CD34^+^ cells (without T cells) were also transplanted into separate animals as negative xGVHD controls. Once again, we found that Minnelide treatment significantly prevented severe GVHD in this xenograft model, as assessed by weight loss, clinical score, and overall survival (Figure 8, B–E). Notably, donor human CD4^+^ and CD8^+^ T cells were almost completely absent from the blood of Minnelide-treated animals, similar to the negative control mice. In contrast, mice undergoing xGVHD contained a marked frequency of human donor cells in the peripheral blood (Figure 8F). Interestingly, at this time, increased levels of donor CD14^+^ cells were identified in these animals, consistent with the notion that Minnelide did not directly damage this lineage (Figure 8F). To directly address triptolide regulation of human T cell proliferation, PBMCs were stimulated in vitro with an anti-CD3 mAb and allogeneic stimulator cells (Figure 8G). Both polyclonal and antigen-stimulated cultures exhibited dose-dependent triptolide inhibition of CD4^+^ and CD8^+^ T cell proliferation. In total, these immune findings demonstrated that Minnelide suppressed xGVHD by specifically targeting human donor T cells.

## Discussion

Immunosuppressants with calcineurin inhibitors remain the standard of care for recipients of aHSCT to prevent GVHD. Unfortunately, these compounds have multiple off-target effects on a variety of tissues. More recently, PTCy has been used as GVHD prophylaxis in matched and mismatched donors with good GVHD prevention, but with its own toxicities (7, 8, 10, 12, 35). GVHD prophylaxis balancing reduction of GVHD and preservation of GVT is still needed. Triptolide compounds have been previously examined for antiinflammatory activity as well as their ability to inhibit immune responses, including GVHD; however, triptolide is largely insoluble in aqueous solvents, limiting its clinical application (14). Ideally, triptolide derivatives that are water soluble with high efficacy and low toxicity would represent an advance for in vivo use to regulate transplant immunity. Minnelide, a water-soluble triptolide prodrug, was shown to exhibit minimal toxicity even after more than 1 year of in vivo administration and it can rapidly convert to triptolide in the presence of phosphatases, which are ubiquitous (29). Preclinical studies reported that Minnelide possesses potent antiproliferative activity against several cancers, including pancreatic and hematologic, dramatically reducing tumor growth, preventing metastasis, and improving overall survival (30, 34, 37). Minnelide is also the most clinically advanced compound for cancer treatment among all triptolide analogs (ClinicalTrials.gov NCT03117920) (17, 38). Here, we demonstrated that low doses of Minnelide treatment prevented severe acute GVHD using multiple models of aHSCT, with substantial improvement in outcomes without abrogating antitumor activity, i.e., GVT responses against experimental AML. Overall, in addition to ameliorating GVHD, Minnelide may be effective in eradicating residual disease in some hematological malignancies due to both direct and indirect (via GVT) activities, thereby providing a potentially novel therapeutic approach to improve aHSCT.

Recently, Giri et al. showed that Minnelide treatment exhibited potent antileukemic effects in human AML in vitro and in vivo models, using tumor cell lines and patient-derived cells (34). Similarly, in the present study, we found that triptolide induced dose-dependent killing of the P815 mastocytoma and A20 B cell lymphoma tumor cell lines in vitro and Minnelide exhibited antitumor activity in vivo (A20). Since the MLL-AF9^GFP^ cells did not show susceptibility in vivo to the Minnelide dose being used, this AML was selected to enable evaluation of GVT activity following aHSCT and results showed that the antitumor response was maintained concurrent with a reduction in GVHD. Therefore, we posit that the potential combination of direct antitumor activity concomitant with GVHD-mediated suppression and maintenance of GVT responses make Minnelide an attractive agent for clinical development.

Two decades ago, triptolide and its derivatives were examined in murine haploidentical MHC (B6→F_1_) and MHC-matched (B10.D2→BALB/c) aHSCT models (19, 20). Using a water-soluble triptolide derivative (PG490-88, 0.535 mg/kg for 21 days), Chen et al. found amelioration of preclinical GVHD and proposed that it involved inhibition of early IL-2 synthesis (19). Because Vβ3^+^ T cell deletion was incomplete, the authors implicated antigen-specific tolerance. In vitro, triptolide was previously reported to inhibit NF-κB, NFAT, and diminished IL-2 production in T cells (39). In our studies, Minnelide was employed at a substantially lower concentration (0.1 mg/kg) to investigate its capacity to inhibit initial responses driving GVHD. Indeed, at this low dose, we observed a marked decrease in donor T cell frequency within the peripheral lymphoid compartment following both MHC-mismatched allogeneic (B6→BALB/c) and xenogeneic (HuPBMC→NSG) transplantation as early as 1–3 weeks after aHSCT (Figure 1, Figure 5, and Figure 8). Consistent with the diminished xGVHD findings, triptolide diminished human T cell proliferation in response to anti-TCR and alloantigen stimulation. Minnelide also markedly diminished proinflammatory effector populations (CD8^+^ and Th1 IFN-γ–producing cells) and serum levels of proinflammatory cytokines (including TNF-α, IFN-γ, MCP-1, and GM-CSF) (Figures 3 and 4). These findings support the notion that Minnelide abrogates T cell proliferation and inflammatory responses that promote acute GVHD. GVHD prophylaxis for patients often involves calcineurin inhibitor–based (CNI-based) regimens with methotrexate with or without anti–thymocyte globulin for HLA-matched transplants (40). PTCy plus tacrolimus with or without mycophenolate mofetil (MMF) has been introduced for haplo-mismatched transplants and shown promise compared with tacrolimus ± MMF ± methotrexate based on improvement reported in a number of studies, including lower grade 3–4 GVHD and diminished lower GI and chronic GVHD versus CNI (41). Since 2017, several new drugs have been FDA approved for second-line defense, i.e., ibrutinib, belumosudil, and ruxolitinib, and the CTLA-4 costimulation blocker abatacept is currently being examined for GVHD treatment (42–45). While CNI-associated toxicity in elderly patients can include nephrotoxicity, hypertension, and electrolyte abnormalities, they are administered typically for several (often 3–6) months and can be tapered (46, 47). Studies here found that 14 days of Minnelide treatment in MHC-mismatched recipients resulted in long-lasting effects without loss of antitumor activity. As noted above, we also observed that triptolide in vitro and Minnelide in vivo exhibited antitumor activity, suggesting the drug could provide additional benefit to patients transplanted due to hematopoietic cancers. Therefore, it may be interesting to consider combining CNI with Minnelide, as both inhibit T cells, and Minnelide also elevated regulatory populations.

One week after transplantation, together with the diminished cytokine levels noted above, a marked reduction in donor T cell infiltrate was found in the colon of Minnelide-treated recipients. Strikingly, this effect was maintained 8 weeks after transplantation and was accompanied by conserved villi and overall improved tissue architecture compared with untreated animals. Early damage to the GI tract results in leakage of bacterial products, including LPS, which plays a key role in driving intestinal inflammation and development of GVHD. Following LPS binding to TLR4, MyD88 signaling leads to NF-κB activation. Along with other transcription factors, these events promote cytokine/chemokine production contributing to initiation and amplification of systemic inflammation, resulting in acute GVHD that is often associated with morbidity and mortality after aHSCT (48, 49). We previously reported that Minnelide treatment downregulated NF-κB activity in pancreatic tumors (37). Notably, experiments here showed Minnelide significantly inhibited NF-κB activation in vivo following LPS administration. Altogether, these findings demonstrated that Minnelide prevented GVHD-driven events, including GI T cell infiltration, intestinal tissue damage, and inflammatory cytokine production. We propose that the latter process might be mediated by Minnelide’s inhibition of NF-κB signaling.

Triptolide and its analogs have been found to regulate several immune cell populations in pathologic conditions. For example, in rheumatoid arthritis, triptolide suppressed maturation, migration, and differentiation of DCs (50, 51). Triptolide was also reported to diminish costimulatory molecules and inflammatory cytokines in monocytes/macrophages (14, 52). These findings, together with the decrease in inflammatory cytokine serum levels in Minnelide-treated animals early after aHSCT, makes it tempting to speculate that the compound could also affect function and migration of APCs in transplanted mice. In physiologic conditions, we found that Minnelide treatment did not affect B cells, CD4^+^ and CD8^+^ T cells, or Tregs in naive mice. Regarding the latter, p-STAT5 expression, which is required for Treg proliferation and function, was not altered by Minnelide treatment in animals undergoing Treg expansion, supporting the notion that Treg IL-2 signaling remained intact (data not shown). Interestingly, in the setting of GVHD after donor T cells were transplanted, an increase in CD4^+^FoxP3^+^ Treg frequency was observed with Minnelide treatment in several GVHD target tissues (e.g., spleen, colon, and lung). Remarkably, with regard to a regulatory cell mechanism, 3 weeks after transplantation in treated recipients, several innate regulatory populations were also evaluated. Increased levels of MDSCs and ILC2s, with the latter population including significant expression of ICOS that reportedly regulates these cells under homeostatic and inflammatory conditions in mice (53), were identified. Thus, we posit that together with direct inhibition of T cell proliferation, Minnelide’s induction of an adaptive and innate regulatory cell network may account for GVHD suppression.

In total, findings in the present study demonstrated Minnelide regulation of mouse as well as human T cell immune responses in the aHSCT setting. Notably, this compound is currently in clinical trials for patients with gastric/pancreatic cancers (ClinicalTrials.gov NCT01927965 and NCT03117920) and a study is now recruiting for treatment of non–transplant-related relapsed or refractory AML (ClinicalTrials.gov NCT03760523). Graft-versus-malignancy responses following aHSCT are crucial for the eradication of hematologic malignancies, particularly AML (3). The experimental findings here using a murine leukemia model are encouraging, as reduction in GVHD was accompanied by GVT responses against this AML. Dependent on the concentration of Minnelide, such responses may benefit from its direct antitumor activity in certain cancers. Findings here indicated that in comparison with PTCy application, Minnelide treatment resulted in equivalent reduction in GVHD and improvement of outcomes. Overall, we propose that Minnelide treatment may provide a new translational therapeutic strategy for patients undergoing aHSCT for hematologic cancers to reduce GVHD and maintain GVT.

## Methods

### Sex as biological variable.

The nature of the experiments involves transplants in which cages must be maintained for extended time periods. The experimental results were therefore obtained using female animals without bias from injuries and hormones due to fighting and aggressive behavior in the cages. We anticipate that results would be the same in males.

### Animals.

C57BL/6J (B6, stock 000664), BALB/c (stock 000651), C3H.SW (stock 000438), B6-CD45.1 breeder (stock 002014), B6-EGFP breeder (stock 003291), NGL-NF-κB-GFP-luciferase [FVB.Cg-Tg(HIV-EGFP,luc)8Tsb/J; stock 027529], and NSG (stock 005557) mice were purchased from The Jackson Laboratory and maintained in our animal facility. All mice were maintained in specific pathogen–free housing at the University of Miami and given autoclaved food and water ad libitum. Mice were used at 8–16 weeks of age.

### aHSCT and Minnelide treatment.

For MHC-mismatched transplants, BALB/c mice received 7.5–8.5 Gy on day −1 and BM was injected 24 hours later with or without T cells from sex- and age-matched B6 (5.5 × 10^6^ BM cells after T cell depletion [TCD] and pooled splenocytes containing 0.8 × 10^6^ T cells) donors. TCD of the BM was performed using HO134 hybridoma supernatant (anti-Thy1.2, 40% of final volume at 25 × 10^6^ cells/mL) and rabbit complement (Cedarlane Labs) immediately prior to transplantation. Mice were monitored 3 times per week for weight loss and clinical score, as previously described (54, 55). In brief, clinical signs of GVHD were scored on a scale from 0 to 2 for 5 parameters: weight loss, diarrhea, fur texture, posture, and alopecia. Mice exhibiting a clinical score greater than 6 were sacrificed and their death was recorded as the next day, in accordance with our animal protocols. Minnelide was dissolved in saline and administered intraperitoneally at a dose of 0.05 mg/kg 2 times per day or 0.1 mg/kg/day for 30 consecutive days.

Experiments to assess GVT utilized MLL-AF9 GFP^+^ BALB/c leukemia cells (56). For these experiments, 5000 leukemia cells were cotransplanted with B6 donor spleen with and without TCD BM cells. Tumor burden was assessed via flow cytometric analysis of blood, spleen, and BM compartments.

For a description of histopathology and immunostaining, allogeneic heterotopic skin transplantation, flow cytometry, multiplex cytokine array, and T cell proliferation assay, see Supplemental Methods. A complete list of antibodies used in this study can be found in Supplemental Table 1.

### Xenogeneic human to mouse transplantation and Minnelide treatment.

HuPBMCs were isolated from human mobilized (Filgrastim) peripheral blood by Ficoll separation and viable T cells counted. NSG mice were irradiated (2 Gy, total body irradiation) and transplanted the following day with 6 × 10^6^ HuPBMCs, which included 3.6 × 10^6^ T cells and approximately 2 × 10^4^ CD34^+^ cells. Recipient mice were injected with or without 0.1 mg/kg Minnelide from day –2 to day 28. Mice were monitored 3 times per week for GVHD clinical score (as above), weight loss, and survival until 6 weeks after transplantation. Control recipients were administered positively selected human CD34^+^ cells from the same donor to mimic BM-only groups in the aHSCT.

### One-way mixed lymphocyte reaction.

Fresh blood (30 to 40 mL) from 2 genetically distinct donors was collected and HuPBMCs were isolated using Ficoll-Paque (GE Healthcare) following the manufacturer’s protocol. Triptolide (Sigma-Aldrich) stock, 1 mM in DMSO, was added to complete media to a concentration of 12.5 nM followed by serial dilutions to 2.5, 0.5, and 0.1 nM. The vehicle was DMSO added to complete media at a concentration of 12.5 nM. Isolated HuPBMCs were plated at 500,000 responder and 1,000,000 irradiated stimulator HuPBMCs per well. Responder HuPBMCs were labeled with CellTrace Violet (CTV; Thermo Fisher Scientific) on day 0 of the culture at 2.5 μM CTV final concentration prior to plating. Wells were collected on day 6 for manual counting and CTV proliferation assessment gating CD4^+^ and CD8^+^ lymphocytes. For in vitro stimulation, PBMCs isolated from 1 donor were plated in wells with OKT3 (1 μg/well) and rhIL-2 (200 ng/well) at 1,000,000 HuPBMCs/well after CTV labeling on day 0 at 2.5 μM CTV final concentration. Wells were collected on day 4 for manual counting and CTV proliferation assessment after gating on CD8^+^ lymphocytes.

### Statistics.

Numbers of animals per group are described in the figure legends. All figure panels include data sets obtained from individual animals. All graphing and statistical analyses were performed using GraphPad Prism 9. Significance of differences between 2 experimental groups was determined using a 2-tailed, unpaired *t* test. For experiments comparing more than 2 groups, data were analyzed using a 1-way or 2-way ANOVA with a post hoc Dunnett’s or Tukey’s multiple-comparison test. For experiments using multiple *t* tests over time, *P* values were adjusted using the 2-stage linear step-up procedure of Benjamini, Krieger, and Yekutieli (57). For survival analyses, a log-rank (Mantel-Cox) test was performed. Statistical tests performed are indicated in the figure legends. A *P* value of less than 0.05 was considered significant: *P < 0.05, **P < 0.01, ***P < 0.001, ****P < 0.0001. Data are shown as mean ± SEM.

### Study approval.

All animal procedures used were performed under protocols approved by the University of Miami Institutional Animal Care and Use Committee (IACUC protocol numbers 19–114 and 18–036; Coral Gables, Florida, USA). All human cells were obtained from consented donors according to IRB-approved protocol 20160363.

### Data availability.

Data can be accessed from the corresponding author (RBL) upon request. All raw data supporting the conclusions of this paper can be found in the supplemental Supporting Data Values file.

## Author contributions

SNC and VTG designed research studies, discussed, analyzed and interpreted data, conducted experiments, and wrote the manuscript. HB and BMV conducted experiments and analyzed data. CSB, DW, BP, MG, and AVV performed research, analyzed and interpreted data, and edited the manuscript. KVK, CLB, and SP provided reagents and edited the manuscript. AKS discussed data and supported the research. RBL designed experiments, discussed, analyzed and interpreted data, wrote the manuscript, and supervised and supported the research.

## Supplementary Material

Supplemental data

Supporting data values

## Version 1 04/11/2024

In-Press Preview

## Version 2 05/08/2024

Electronic publication

## References

[1] Appelbaum FR (2001). Haematopoietic cell transplantation as immunotherapy. Nature.

[2] Zeiser R, Blazar BR (2017). Acute graft-versus-host disease - biologic process, prevention, and therapy. N Engl J Med.

[3] Horowitz MM (1990). Graft-versus-leukemia reactions after bone marrow transplantation. Blood.

[4] Ferrara JL, Reddy P (2006). Pathophysiology of graft-versus-host disease. Semin Hematol.

[5] Ferrara JL (1999). Pathophysiologic mechanisms of acute graft-vs.-host disease. Biol Blood Marrow Transplant.

[6] Welniak LA (2007). Immunobiology of allogeneic hematopoietic stem cell transplantation. Annu Rev Immunol.

[7] Ahmed S (2019). Lower graft-versus-host disease and relapse risk in post-transplant cyclophosphamide-based haploidentical versus matched sibling donor reduced-intensity conditioning transplant for Hodgkin lymphoma. Biol Blood Marrow Transplant.

[8] Shaw BE (2021). National marrow donor program-sponsored multicenter, phase II trial of HLA-mismatched unrelated donor bone marrow transplantation using post-transplant cyclophosphamide. J Clin Oncol.

[9] Ganguly S (2014). Donor CD4^+^ Foxp3^+^ regulatory T cells are necessary for posttransplantation cyclophosphamide-mediated protection against GVHD in mice. Blood.

[10] Robinson TM (2016). Haploidentical bone marrow and stem cell transplantation: experience with post-transplantation cyclophosphamide. Semin Hematol.

[11] Mielcarek M (2016). Posttransplantation cyclophosphamide for prevention of graft-versus-host disease after HLA-matched mobilized blood cell transplantation. Blood.

[12] Ciurea SO (2015). Haploidentical transplant with posttransplant cyclophosphamide vs matched unrelated donor transplant for acute myeloid leukemia. Blood.

[13] Tao X (1991). Effect of an extract of the Chinese herbal remedy Tripterygium wilfordii Hook F on human immune responsiveness. Arthritis Rheum.

[14] Yuan K (2019). Application and mechanisms of triptolide in the treatment of inflammatory diseases-a review. Front Pharmacol.

[15] Phillips PA (2007). Triptolide induces pancreatic cancer cell death via inhibition of heat shock protein 70. Cancer Res.

[16] Chen X (2005). Triptolide, a constituent of immunosuppressive Chinese herbal medicine, is a potent suppressor of dendritic-cell maturation and trafficking. Blood.

[17] Noel P (2019). Triptolide and its derivatives as cancer therapies. Trends Pharmacol Sci.

[18] Qiu D, Kao PN (2003). Immunosuppressive and anti-inflammatory mechanisms of triptolide, the principal active diterpenoid from the Chinese medicinal herb Tripterygium wilfordii Hook. f. Drugs R D.

[19] Chen BJ (2000). Prevention of graft-versus-host disease by a novel immunosuppressant, PG490-88, through inhibition of alloreactive T cell expansion. Transplantation.

[20] Fidler JM (2002). Immunosuppressive activity of the Chinese medicinal plant Tripterygium wilfordii. III. Suppression of graft-versus-host disease in murine allogeneic bone marrow transplantation by the PG27 extract. Transplantation.

[21] Chen BJ (2002). Mechanisms of tolerance induced by PG490-88 in a bone marrow transplantation model. Transplantation.

[22] Tang W (2005). Prevention of graft-versus-host disease by a novel immunosuppressant, (5R)-5-hydroxytriptolide (LLDT-8), through expansion of regulatory T cells. Int Immunopharmacol.

[23] He HT (2019). The immunomodulatory effect of triptolide on mesenchymal stem cells in vitro. Blood.

[24] Chen Y (2000). PG27, an extract of Tripterygium wilfordii Hook F, induces antigen-specific tolerance in bone marrow transplantation in mice. Blood.

[25] Li ZY (2013). Prevention of acute GVHD in mice by treatment with Tripterygium hypoglaucum Hutch combined with cyclosporin A. Hematology.

[26] Wu XL (2020). Chinese medicine treatment on graft-versus-host disease after allogeneic hematopoietic stem cell transplantation. Chin J Integr Med.

[27] Asano K (1997). Inhibition of murine chronic graft-versus-host disease by the chloroform extract of Tripterygium wilfordii Hook f. Transpl Immunol.

[28] Xu L (2013). Acute and subacute toxicity studies on triptolide and triptolide-loaded polymeric micelles following intravenous administration in rodents. Food Chem Toxicol.

[29] Chugh R (2012). A preclinical evaluation of Minnelide as a therapeutic agent against pancreatic cancer. Sci Transl Med.

[30] Banerjee S, Saluja A (2015). Minnelide, a novel drug for pancreatic and liver cancer. Pancreatology.

[31] Rousalova I (2013). Minnelide: a novel therapeutic that promotes apoptosis in non-small cell lung carcinoma in vivo. PLoS One.

[32] Isharwal S (2017). Minnelide inhibits androgen dependent, castration resistant prostate cancer growth by decreasing expression of androgen receptor full length and splice variants. Prostate.

[33] Banerjee S (2013). Minnelide reduces tumor burden in preclinical models of osteosarcoma. Cancer Lett.

[34] Giri B (2019). Pre-clinical evaluation of Minnelide as a therapy for acute myeloid leukemia. J Transl Med.

[35] Luznik L, Fuchs EJ (2010). High-dose, post-transplantation cyclophosphamide to promote graft-host tolerance after allogeneic hematopoietic stem cell transplantation. Immunol Res.

[36] Ross D (2013). Antigen and lymphopenia-driven donor T cells are differentially diminished by post-transplantation administration of cyclophosphamide after hematopoietic cell transplantation. Biol Blood Marrow Transplant.

[37] Banerjee S (2014). CD133^+^ tumor initiating cells in a syngenic murine model of pancreatic cancer respond to Minnelide. Clin Cancer Res.

[38] Liu Y (2013). Low-dose triptolide in combination with idarubicin induces apoptosis in AML leukemic stem-like KG1a cell line by modulation of the intrinsic and extrinsic factors. Cell Death Dis.

[39] Qiu D (1999). Immunosuppressant PG490 (triptolide) inhibits T-cell interleukin-2 expression at the level of purine-box/nuclear factor of activated T-cells and NF-kappaB transcriptional activation. J Biol Chem.

[40] Storb R (1989). Methotrexate and cyclosporine versus cyclosporine alone for prophylaxis of graft-versus-host disease in patients given HLA-identical marrow grafts for leukemia: long-term follow-up of a controlled trial. Blood.

[41] Saliba RM (2022). Characteristics of graft-versus-host disease (GvHD) after post-transplantation cyclophosphamide versus conventional GvHD prophylaxis. Transplant Cell Ther.

[42] Zeiser R, Lee SJ (2022). Three US Food and Drug Administration-approved therapies for chronic GVHD. Blood.

[43] Watkins B (2021). Phase II trial of costimulation blockade with abatacept for prevention of acute GVHD. J Clin Oncol.

[44] Przepiorka D (2022). FDA approval summary: belumosudil for adult and pediatric patients 12 years and older with chronic GvHD after two or more prior lines of systemic therapy. Clin Cancer Res.

[45] Martini DJ (2022). Recent FDA approvals in the treatment of graft-versus-host disease. Oncologist.

[46] Ratanatharathorn V (1998). Phase III study comparing methotrexate and tacrolimus (prograf, FK506) with methotrexate and cyclosporine for graft-versus-host disease prophylaxis after HLA-identical sibling bone marrow transplantation. Blood.

[47] Kanakry CG (2017). Low immunosuppressive burden after HLA-matched related or unrelated BMT using posttransplantation cyclophosphamide. Blood.

[48] Hill GR, Ferrara JL (2000). The primacy of the gastrointestinal tract as a target organ of acute graft-versus-host disease: rationale for the use of cytokine shields in allogeneic bone marrow transplantation. Blood.

[49] Ferrara JL (2009). Graft-versus-host disease. Lancet.

[50] Liu Q (2007). Triptolide impairs dendritic cell migration by inhibiting CCR7 and COX-2 expression through PI3-K/Akt and NF-kappaB pathways. Mol Immunol.

[51] Zhu KJ (2005). Triptolide affects the differentiation, maturation and function of human dendritic cells. Int Immunopharmacol.

[52] Liu J (2005). Triptolide suppresses CD80 and CD86 expressions and IL-12 production in THP-1 cells. Acta Pharmacol Sin.

[53] Paclik D (2015). ICOS regulates the pool of group 2 innate lymphoid cells under homeostatic and inflammatory conditions in mice. Eur J Immunol.

[54] Wolf D (2017). Marked in vivo donor regulatory T cell expansion via interleukin-2 and TL1A-Ig stimulation ameliorates graft-versus-host disease but preserves graft-versus-leukemia in recipients after hematopoietic stem cell transplantation. Biol Blood Marrow Transplant.

[55] Cooke KR (1996). An experimental model of idiopathic pneumonia syndrome after bone marrow transplantation: I. The roles of minor H antigens and endotoxin. Blood.

[56] Zhang J (2015). ST2 blockade reduces sST2-producing T cells while maintaining protective mST2-expressing T cells during graft-versus-host disease. Sci Transl Med.

[57] Benjamini Y (2006). Adaptive linear step-up procedures that control the false discovery rate. Biometrika.

