# Supplementary material for: Minnelide suppresses GVHD and enhances survival while maintaining GVT responses
Source: JCI Insight. 2024 Apr 11;9(9):e165936. doi: 10.1172/jci.insight.165936 (PMC11141936; doi:10.1172/jci.insight.165936)
Supplement: Supplemental data [file jciinsight-9-165936-s227.pdf]

# *Supplementary Material*

## Supplementary Methods

### Flow cytometric analyses

At the indicated time points, after HSCT, peripheral blood, spleen, or pooled lymph nodes (mesenteric, inguinal, brachial, axillary, and cervical) were collected from transplant recipients. Lymphoid organs were prepared into single-cell suspensions and filtered through 200-μm nylon mesh, and peripheral blood mononuclear cells were separated using a standard Ficoll-Paque (GE Healthcare) density separation. For surface antigens, single-cell suspensions were stained with fluorescently labeled antibodies (purchased from BD, Biosciences or BioLegend) directly ex vivo in phosphate-buffered saline (PBS) supplemented with 2% fetal bovine serum and 0.1% sodium azide. When needed, cells were fixed with the Foxp3/Transcription Factor Staining Buffer Set (eBiosciences) for all stains as per the manufacturer's instructions, and then were stained with intracellular antibodies. For cytokine staining, single-cell suspensions were cultured with immobilized anti-mouse CD3ε antibody for 16 hours (clone: 17A2; BioXcell, USA) and brefeldin A (10 μg/ml) was added to all cultures for the final 3 h (Sigma-Aldrich, USA). Cells were stained with extracellular antibodies, fixed with the BD Cytofix/Cytoperm Fixation and Perm/Wash buffer as per the manufacturer's instructions, and then stained with intracellular antibodies. Samples were acquired on a LSR Fortessa (BD Biosciences) and Aurora (Cytek Biosystems, USA) flow cytometers, and analysis was performed using FlowJo software (v.10.4.1, TreeStar) using single-color compensation controls and fluorescence-

minus-one thresholds to set gate margins. Antibodies utilized in this study are described in Table S1.

**Allogeneic heterotopic skin transplantation**

Allograft dorsal ear skin from euthanized F1 (B6×BALB/c, MHC H-2Kb/d) mice and C3H (MHC H-2Kk) mice was transplanted onto a 0.5 × 0.5 cm2 prepared left lateral thoracic cavity skin bed of isoflurane-anesthetized BALB/c mice 3 months after B6 allogenic HSCT (MHC H-2Kb/d alleles). The F1 skin allografts were placed cephalad and ventral and the C3H skin allografts caudal and dorsal at the prepared skin bed site. The site was covered with an adhesive bandage, which remained in place for 6 days. Allograft appearance and integrity was assessed subsequently on days 7, 8, 9, 12, 15, 18, 25, and 32. Graft scoring was as follows: 0, intact graft and healthy appearance; 1, inflamed graft, but no signs of necrosis; 2, inflamed graft and less than 25% necrosis; 3, inflamed graft and between 25% and 75% necrosis; and 4, greater than 75% necrosis or loss of graft [35].

**Multiplex cytokine array: CBA immunoassay**

Serum was collected from recipient mice 6 weeks post-aHSCT via cardiac puncture. Quantification of serum cytokines was performed using a LEGENDplex™ Mouse Inflammation Panel (13-plex) and MU Th Cytokine Panel (12-plex) kit according to the manufacturer's instructions (Biolegend). Samples were run on a Beckman Coulter CytoFLEX flow cytometer and analyzed using LEGENDplex Software.

**Histopathology and Immunohistochemistry**

At the conclusion of experiments, colon, ear skin, or interscapular skin were collected, formalin-fixed, embedded in paraffin, sectioned, and stained with H&E. Slides were scored as previously described [35]. Sections were given a pathology score of 0–2 (0=normal, 1=moderate, 2=severe) based upon the amount of inflammation/infiltration, collagen deposition and dermal thickening for skin, and inflammation/infiltration, edema, mucosal thickening, and crypt structure for colon. Scores were then aggregated to calculate an overall histopathology score (max score = 6 for skin, 8 for colon).

For immunohistochemistry, colon sections were deparaffinized followed by heat induced antigen retrieval in citrate buffer (pH = 6.0) and incubated with 3% H2O2 for 10 minutes. Tissues were further immunostained using CD3ε and CD11c antibody (Cell Signaling - E4T1B) at a dilution of 1:200. Primary antibodies were detected using VECTASTAIN Elite ABC peroxidase kit as per the manufacturer's protocol using diaminobenzidine (DAB). Finally, the sections were counterstained with Mayer's hematoxylin and mounted with D.P.X.

**Isolating Lamina Propria lymphocytes from colons for phenotyping cell populations.**

Briefly modified from (Miltenyi Biotec Lamina Propria Dissociation kit). Intestines were removed and placed in HBSS (w/o) in a Petri dish. Feces were cleared by flushing with HBSS(w/o) using a syringe. The intestines were first cut longitudinally and then laterally

into pieces of approximately 0.5 cm length. Tissue pieces were transferred into a 50 ml tube containing 20 ml of **predigestion solution:** (1xHBSS(w/o) containing 5 mM EDTA, 5% fetal bovine serum (FBS), 1 mM DTT). Samples were incubated for 20 minutes at 37 C under continuous rotation. Samples were mixed well and applied to a 100 uM mesh filter placed on a 50 ml collection tube. Lamina propria tissue pieces were transferred into a new 50 ml tube containing 20 ml of fresh predigestion solution and the incubation repeated. Lamina propria pieces were then transferred into a new 50 ml tube containing 20 ml of HBSS (w/o) and incubated for 20 minutes at 37 C under continuous rotation. After a quick vortex, samples were applied to a 100 uM mesh filter placed on a 50 ml collection tube. **A digestion solution** containing 10% FBS Dnase1 @ 0.1 mg/ml and collagenase 3 @ 600 U/ml was prepared and preheated. Intestinal tissue was placed into a gentleMACS C tube containing 3 ml of digestion solution. Samples were incubated for 30 minutes at 37 C under continuous rotation. A C tube was attached upside down onto the sleeve of the gentleMACS Dissociator and the gentleMACS Program "m_intestine_01" run. After a short spin, samples were resuspended in 5 ml of phosphate buffer solution and applied to a 100 um mesh filter. The cell suspension was centrifuged at 300Xg for 10 minutes at room temperature, aspirated and lamina propria lymphocytes resuspended with appropriate buffer and volume for further applications.

### Isolating Lung lymphocytes for phenotyping cell populations

Briefly modified from (Miltenyi Biotec Lung Dissociation kit). Lungs were perfused through the heart with 10 mL of 1X PBS. Lobes were harvested and placed on a petri dish with 1 mL PBS. Lungs were then transferred into GentleMACS C tubes with 3 mL of 1XPBS

containing 2 mg/ml Collagenase Type IV (ThermoFisher, Gibco) and 1 mg/ml DNase (Sigma-Aldrich). The samples were placed on the GentleMACS Dissociator and run on cycle "m_lung_01". Afterwards, they were incubated at 37 C for 30 minutes with quick vortex every 5-minute intervals. Samples were then placed on the GentleMACS Dissociator on cycle "m_lung_02". Lung tissue was then poured over 100uM mesh cell strainer and into a collection tube. Samples were spun for 5 minutes at 1500 RPM and resuspended in 1 mL of ACK lysis buffer for 5 min at room temperature. The ACK buffer was then washed from the cells with 5 mL of 1X PBS. Cells were then resuspended with appropriate buffer and volume for further applications.

**T cell proliferation assay**

Splenocyte T cells were isolated from EGFP mice using Pan T Cell Isolation Kit II per the manufacturer's protocol (Miltenyi Biotec) and plated in triplicate at 80,000 cells per well in a 96 well plate. T cells were activated with 2 µL of pre-washed CD3/CD28 Dynabeads® and treated with 0, 0.5, 1.5, 5, 15, and 50 nM of triptolide. Cells were imaged at 10x magnification in an IncuCyte Zoom Live-content imaging system (Essen Bioscience) at 37 C with 5% CO2. Images were acquired every 1 h for 3 days, with 4 images per well. Data was analyzed using IncuCyte analysis software to detect and quantify cell confluence (phase-contrast), and dead or dying cells (fluorescence) from the same well.

**Allogeneic bone marrow transplantation and Minnelide treatment**

For MHC-matched transplants, B6 mice received 10.5 Gy total body irradiation (30 cGy/min TBI from a Cs137 source) on day 0. Three hours later, irradiated mice were injected intravenously (IV) with T cell-depleted BM with or without T cells from sex and age matched C3H.SW ($7\times10^6$ BM cells and pooled splenocytes containing $2\times10^6$ $CD8^+$ T cells) donors. Mice were monitored 3x per week for weight loss and clinical score. For chronic GVHD transplants BALB/c mice received 7.5 Gy on the day of transplantation and were injected with donor B10.D2 ($5.0x10^6$ non-T cell depleted BM cells and $25x10^6$ non-fractionated splenocytes. Mice were monitored 3x per week for weight loss and clinical score as previously described[35]. Minnelide was dissolved in saline and administered intraperitoneally (i.p.) at a dose of 0.1 mg/kg/day starting at day 40 for 7 consecutive days. To assess the kinetics of treatment with Minnelide in an acute MHC-mismached GVHD model, BALB/c mice received 7.5 Gy on day −1 and BM was injected 24 hours later with or without T cells from sex and age-matched B6 donors ($5.5 \times10^6$ TCD BM cells and splenocytes containing $0.7\times10^6$ T cells)[35]. Minnelide was dissolved in saline and administered intraperitoneally (i.p.) at a dose of 0.1mg/kg in three different injection schedules. One group received Minnelide treatment post-HSCT on D3+D4, another group D-2 through D7 and a third group D-2 thru D14.

**Cyclophosphamide treatment.**

Cyclophosphamide (50 mg/kg) was administered i.p. on day 3 and 4 post-transplant in MHC-mismatched aHSCT using B6/BALB/c donor/recipient model[39,40].

***In vivo* NF-κB activation**

NF-κB reporter mice (NGL) received intraperitoneal injection of LPS (1mg/kg). Minnelide was administered i.p. 24 hours before and 15 min after LPS injection. After 4 hours, D-Luciferin (LUCK-1G, Gold Biotechnology Inc., MO) was prepared at a dose of 15 mg/ml and injected i.p. at 10 μl/g of mouse body weight for bioluminescence measurement by IVIS Spectrum (PerkinElmer, MA, US). Image quantitation and analysis were made using the Living Image Software (PerkinElmer, MA, US). Data is presented as bioluminescent images and quantified as total flux (photons/sec).

**Tumor cell viability assay**

A20 B cell lymphoma (provided by Dr. Robert Negrin, Stanford University, Standford, CA) and P815 mastocytoma cells were seeded in a 96-well plate (2,000 cells/well) and treated with 0, 0.5, 1, 2, 5, 10, 25, and 50 nM of triptolide for 48h. Cell viability was measured using a WST-8 based cell cytotoxicity assay per the manufacturer's protocol (Dojindo) and expressed after normalizing to untreated cells.

**In vivo tumor cell survival and Minnelide treatment**

A20$^{luciferase}$ B cell lymphoma cells ($2x10^6$ and $5x10^6$) were injected i.v. into BALB/c mice and some animals were administered Minnelide (0.1mg/kg/day). Tumor burden was assessed weekly by bioluminescent imaging (IVIS, see above).

## Supplemental Figures

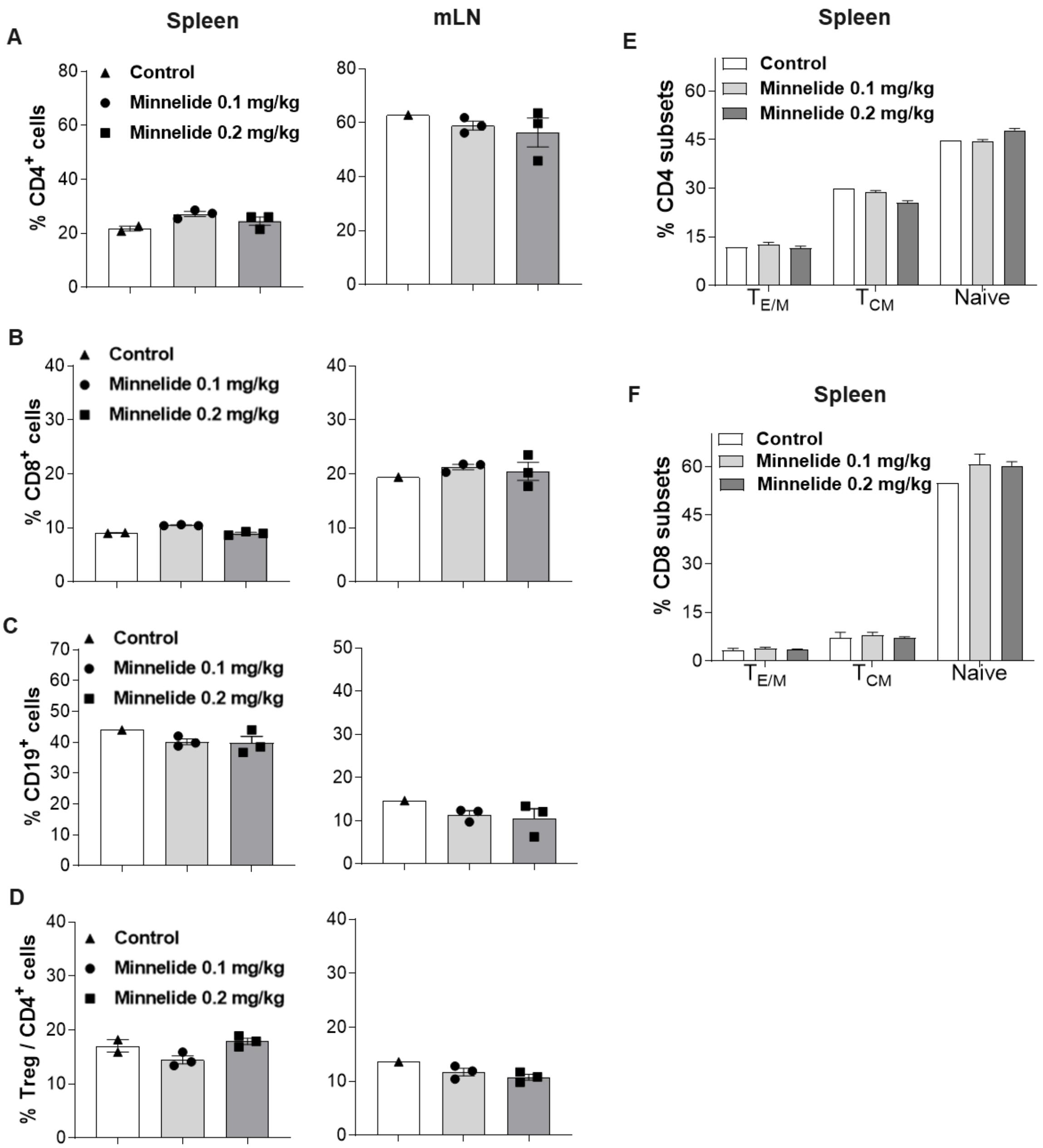


**Figure S1.** ***Daily Minnelide administration for one month does not alter lymphocyte populations in normal mice.*** BALB/c mice were treated with Minnelide (0.1 or 0.2 mg/kg) for 30 days and individual spleen (*left*) and mLN (*right*) were analyzed. Frequency of (**A**) $CD4^+$, (**B**) $CD8^+$, (**C**) $CD19^+$, and (**D**) $CD4^+FoxP3^+$ Tregs were determined. (**E, F**) CD4 and CD8 T cells expressing an effector memory ($T_{E/M}$, $CD44^{hi}CD62L^{lo}$), central memory (TCM $CD44^{hi}CD62L^{hi}$) or naïve ($CD44^{lo}CD62L^{hi}$) phenotype in the spleen. **p<0.01 and *** p<0.001. Data are means ± SEM.

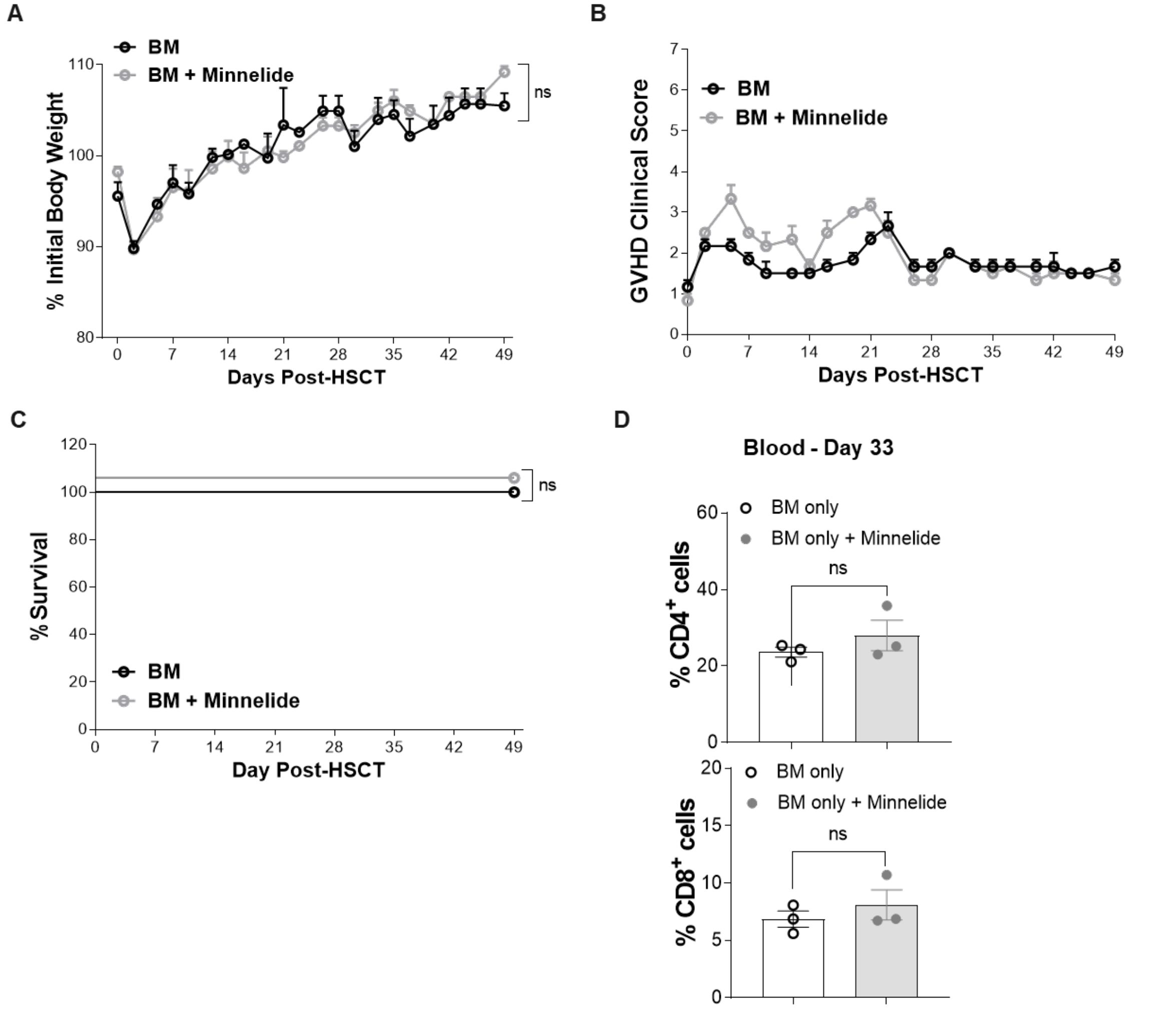


**Figure S2.** ***Minnelide treatment of mice transplanted with allogeneic donor marrow without T cells does not alter outcomes***. (**A-C**) B6 T cell depleted bone marrow (TCD BM) cells were transplanted into BALB/c recipients and Minnelide was administered 1x / day at 0.1mg/kg for one month. (**A**) Weight loss, (**B**) clinical score and (**C**) GVHD overall survival (n=3 mice/group). (**D**) Frequency of $CD4^+$ and $CD8^+$ cells in the blood day 33 post-aHSCT. ns = not statistically significant. Data are means ± SEM.

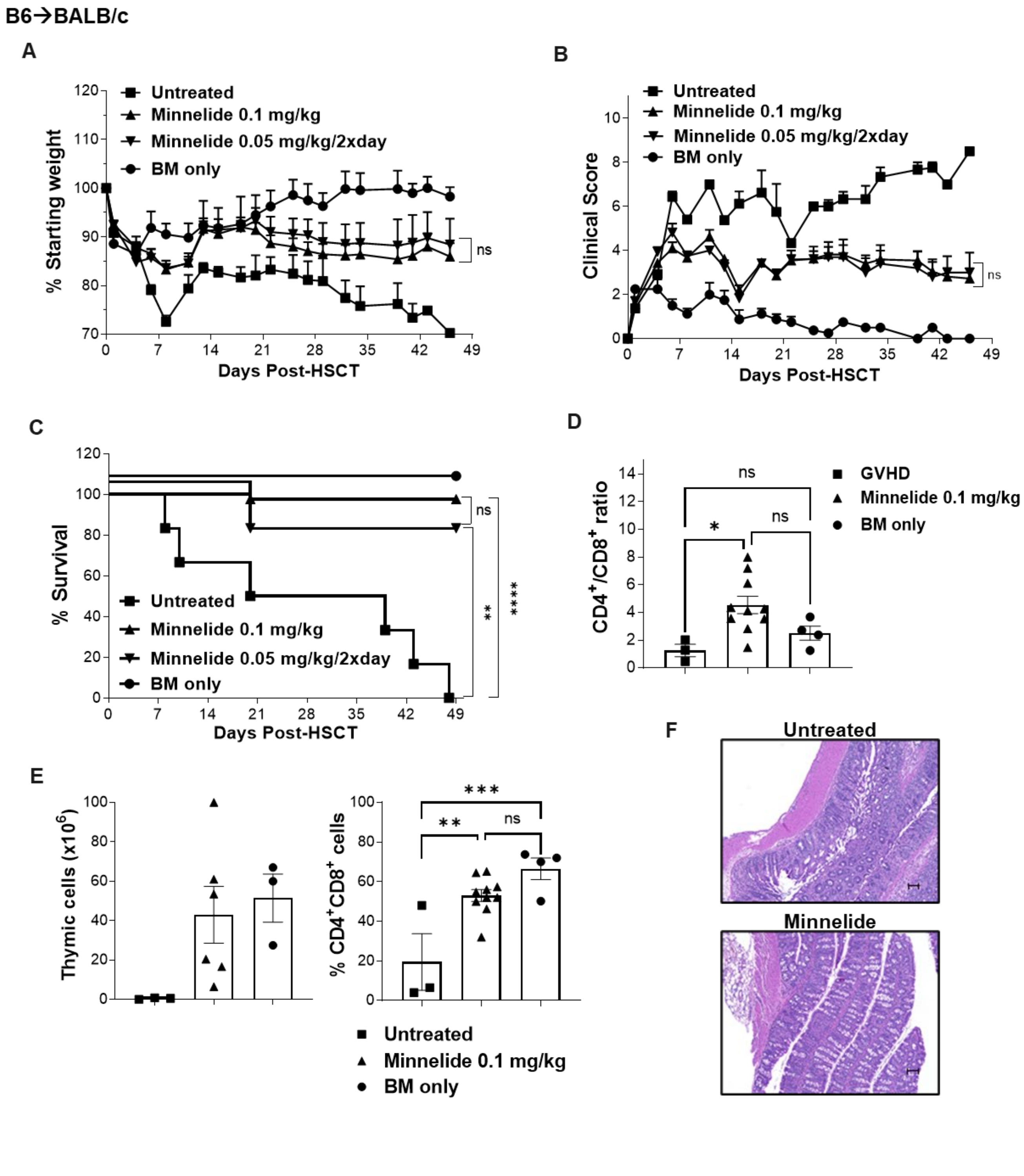


**Figure S3.** ***Administration of a total dose of 0.1mg/kg/day of Minnelide delivered as a single or two injections resulted in the same level of GVHD amelioration.*** BALB/c recipients after transplant of B6 BM and T cells and treated with Minnelide at indicated amounts. (**A**) Weight loss, (**B**) GVHD clinical scores, and (**C**) overall survival of recipient mice (n=6 for untreated, n=16 Minnelide 0.1 mg/kg, n=8 Minelide 0.05 mg/kg,

and n=4 for BM only). (**D**) CD4 to CD8 ratio in the spleen two months post-aHSCT in untreated, treated with Minnelide 0.1 mg/kg/day or BM only mice. (**E**) Number of thymic cells and frequency of donor $CD4^{+}CD8^{+}$ double positive T cells in the thymus of the indicated groups 2 months post-aHSCT (n=3-4 for untreated and BM only group and n=10 for Minnelide-treated group). (**F**) Representative histology (H&E) of colon tissue of untreated or Minnelide-treated recipients 1 week
after transplant. Magnification 100x. Groups were compared using one-way ANOVA with Tukey's multicomparisons test for multiple groups *p<0.05, **p<0.01, *** p<0.001, ns = not statistically significant. Data are means ± SEM.

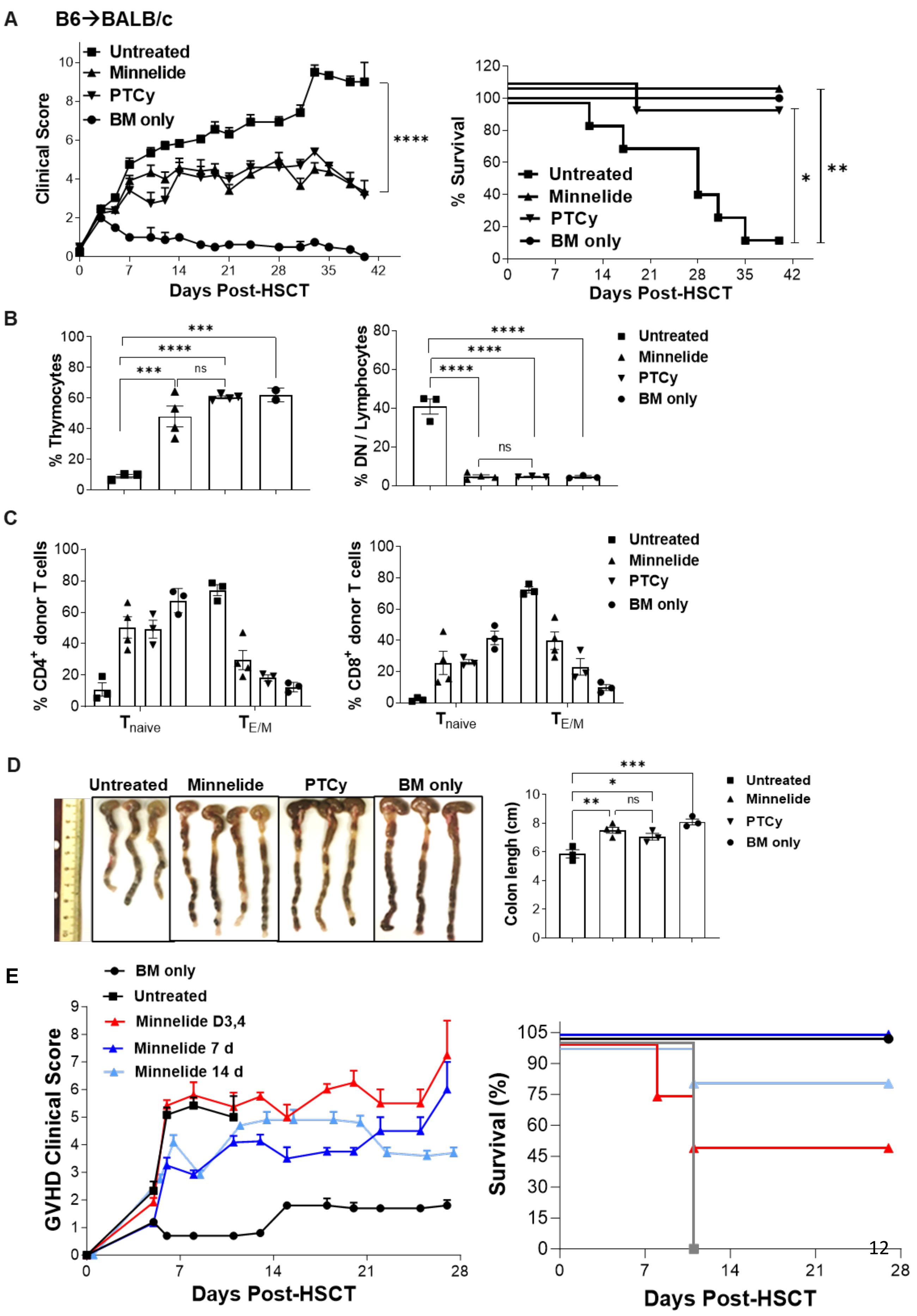
A
B6→BALB/c
Untreated
Minnelide
PTCy
BM only
Clinical Score
Days Post-HSCT
****
% Survival
*
**
B
% Thymocytes
% DN / Lymphocytes
***
****
ns
C
% CD4+ donor T cells
% CD8+ donor T cells
Tnaive
TE/M
D
Colon lengh (cm)
E
BM only
Untreated
Minnelide D3,4
Minnelide 7 d
Minnelide 14 d
GVHD Clinical Score
Survival (%)
Days Post-HSCT

**Figure S4.** ***Recipients treated with Minnelide exhibited similar acute GVHD amelioration compared with PTCy after MHC-mismatched aHSCT.*** Allo-HSCT was performed utilizing a B6 → BALB/c donor and recipient mouse model and recipients were treated with Minnelide 0.1 mg/kg (day −2 to 28) or PTCy (days 3, 4) (n=8 mice/group, n=4 mice BM only group) (**A-D**). (**A**) GVHD clinical scores and overall survival of the indicated groups are shown. (**B**) Frequency of thymocytes and $CD4^-CD8^-$ double negative cells in the thymus of the different groups 6 weeks post-aHSCT. (**C**) Frequency of donor T cells expressing an effector memory ($CD44^{hi}CD62\text{-}L^{lo}$) or naïve ($CD44^{lo}CD62\text{-}L^{hi}$) phenotype of $CD4^+$ (*left*) and $CD8^+$ (*right*) cells in spleens from recipients 6 weeks after transplant. Groups were compared using one-way ANOVA or Two-way ANOVA with Tukey's multi comparisons test. (**D**) Colon length pictures and bar graphs 6 weeks post-aHSCT from the indicated experimental groups. (**E**) Allo-HSCT was performed using a B6→BALB/c donor and recipient mouse model (B6: $0.7\times10^6$ T cells + $5.5\times10^6$ TCD BM cells). Recipients were irradiated with 7.5 Gy the day before transplant and were treated with Minnelide 0.1 mg/kg with three different treatment schedules (day 3, 4), (day -2 through d7) or (d-2 through d14). GVHD clinical scores and overall survival of the indicated groups are shown. (n=6 mice/group, n=5 mice BM only group). Groups were compared using two-tailed unpaired t test or one-way ANOVA with Tukey's multiple comparisons test for multiple groups. *p<0.05, **p<0.01, ***p<0.001, ****p<0.0001. Data are means ± SEM.

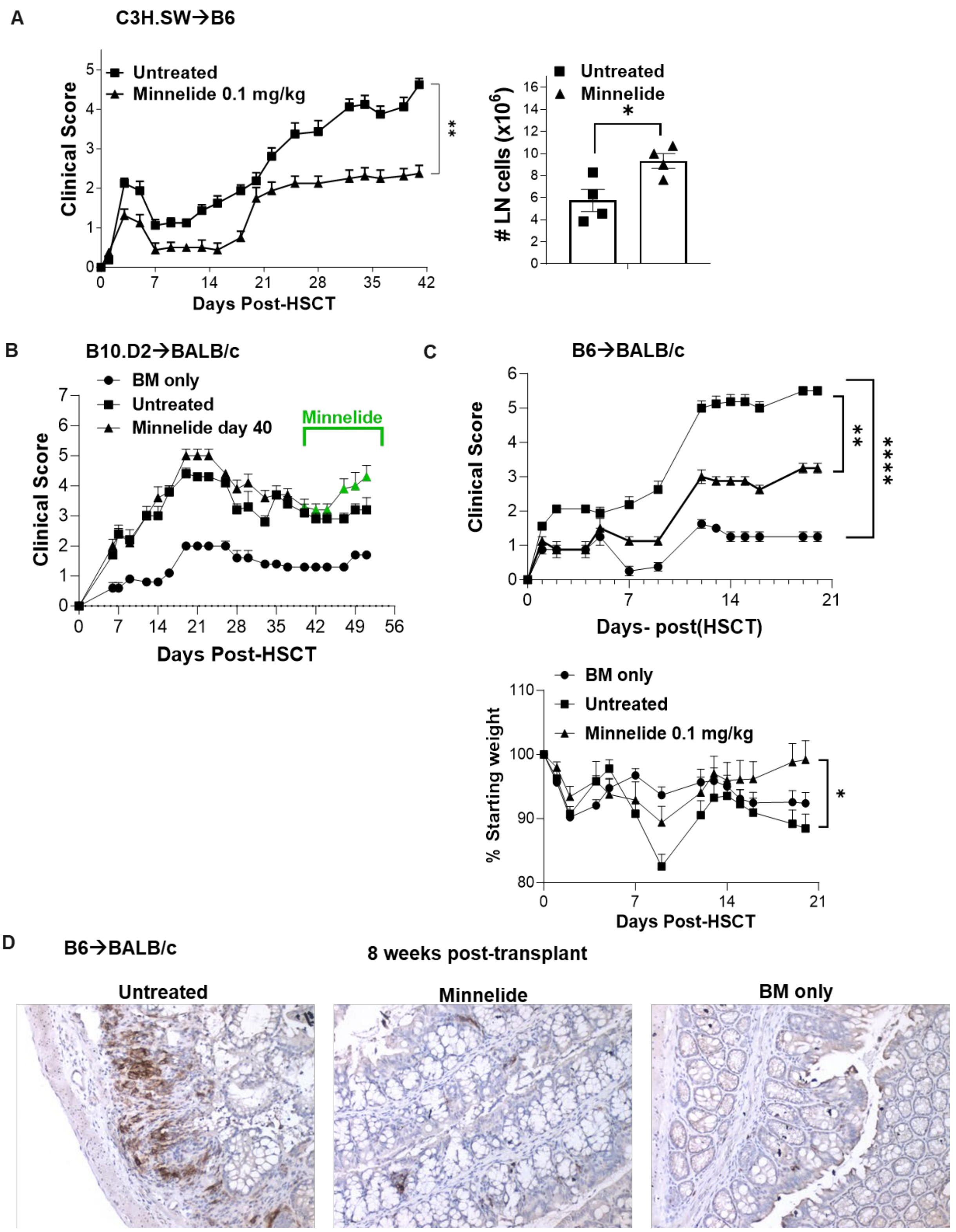
A
C3H.SW→B6
Untreated
Minnelide 0.1 mg/kg
Clinical Score
Days Post-HSCT
**
Untreated
Minnelide
# LN cells (x10^6)
*
B
B10.D2→BALB/c
BM only
Untreated
Minnelide day 40
Minnelide
Clinical Score
Days Post-HSCT
C
B6→BALB/c
Clinical Score
Days- post(HSCT)
**
****
BM only
Untreated
Minnelide 0.1 mg/kg
% Starting weight
Days Post-HSCT
*
D
B6→BALB/c
8 weeks post-transplant
Untreated
Minnelide
BM only

**Figure S5.** ***Recipients treated with Minnelide exhibited acute GVHD amelioration in an MHC-match model but not in chronic GVHD.*** (**A**) Allo-HSCT was performed using a C3H.SW→ B6 donor and recipient mouse model (C3H.SW: $2\times10^6$ $CD8^+$T cells + $7\times10^6$ TCD BM cells). Recipients were irradiated with 10.5 Gy the day of transplant and were treated with Minnelide 0.1 mg/kg (day −1 to 29). GVHD Clinical score (*left*) and number of pooled cervical, axillary, inguinal and mesenteric lymph node cells (*right*) of B6 recipients 6 weeks post-aHSCT (n=8 mice/group). (**B**) Allo-HSCT was performed using a B10.D2→BALB/c donor and recipient mouse model (B10.D2: $25\times10^6$ T unfractionated splenocytes + $5.0\times10^6$ non-TCD BM cells). Recipients were irradiated with 7.5 Gy the day of transplant and were treated with Minnelide therapeutically. Minnelide was dissolved in saline and administered intraperitoneally (i.p.) at a dose of 0.1 mg/kg/day starting at day 40 for 7 consecutive days. (**C**) Allo-HSCT was performed utilizing a B6→BALB/c donor and recipient mouse model and recipients were treated with Minnelide 0.1 mg/kg from day −2 to 20 after transplant, clinical scores (upper panel) and weight loss (lower panel) are presented (n=8 mice untreated, n=4 mice Minnelide and n=4 mice BM only group). (**D**) Minnelide treatment is associated with diminished levels of $CD11c^+$ cells in colonic tissue. Eight weeks post B6→BALB/c aHSCT, representative colons from BM+T untreated (left panel), BM+T Minnelide treated (middle panel) and BM only (right panel) recipients were stained for CD11c expression. Levels of positively stained cells were reduced in the Minnelide treated compared to untreated and BM only recipients. Groups were compared using two-tailed unpaired t test or one-way ANOVA with Tukey's multiple comparisons test for multiple groups. *$p<0.05$, **$p<0.01$, ***$p<0.001$, ****$p<0.0001$. Data are means ± SEM.

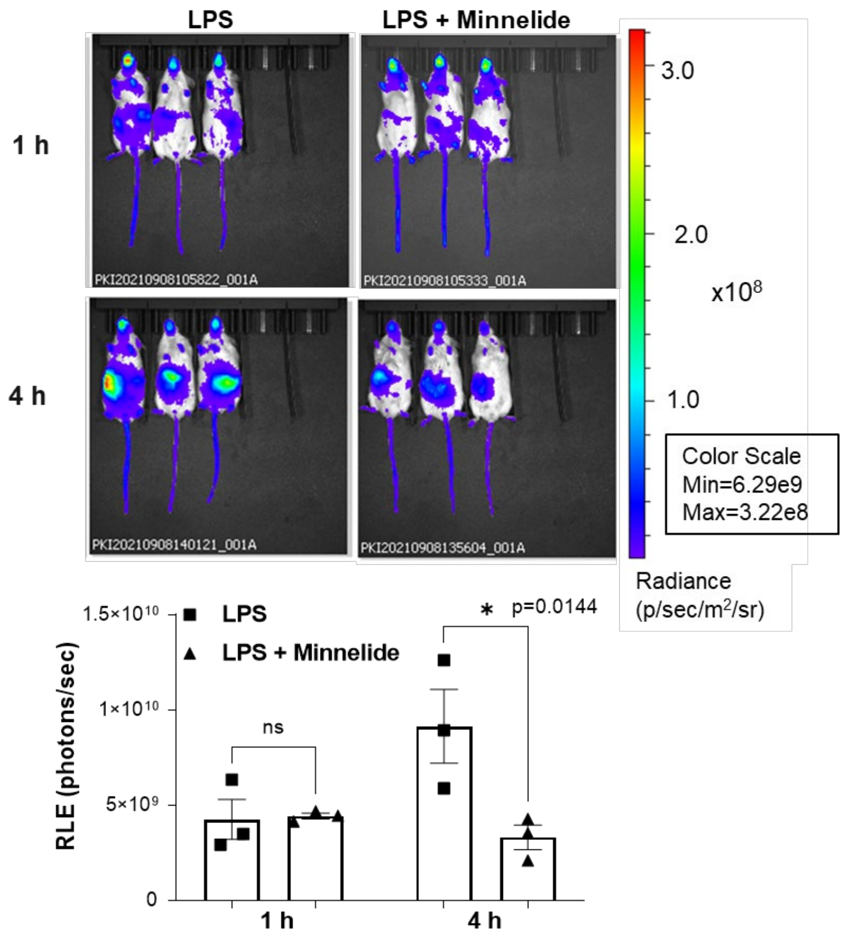


***Figure S6. Minnelide diminishes NFkB activation by 4 hrs. after LPS administration.*** FVB.Cg-Tg (HIV-EGFP,luc)8Tsb/J NGL mice were injected i.p. with 1 mg/kg LPS with or without Minnelide 0.1 mg/kg (n=3 mice/group). NFκB activation was identified by bioluminescence imaging (upper panel) and quantified by measurement of the total photon flux (RLE, relative light emission) from each mouse (bottom panel) 1- and 4-hours post-treatment. Color bar represents signal intensity code over body surface area. Data are means ± SEM. Groups were compared using two-way ANOVA with Bonferroni's multiple comparison test. *$p<0.05$.

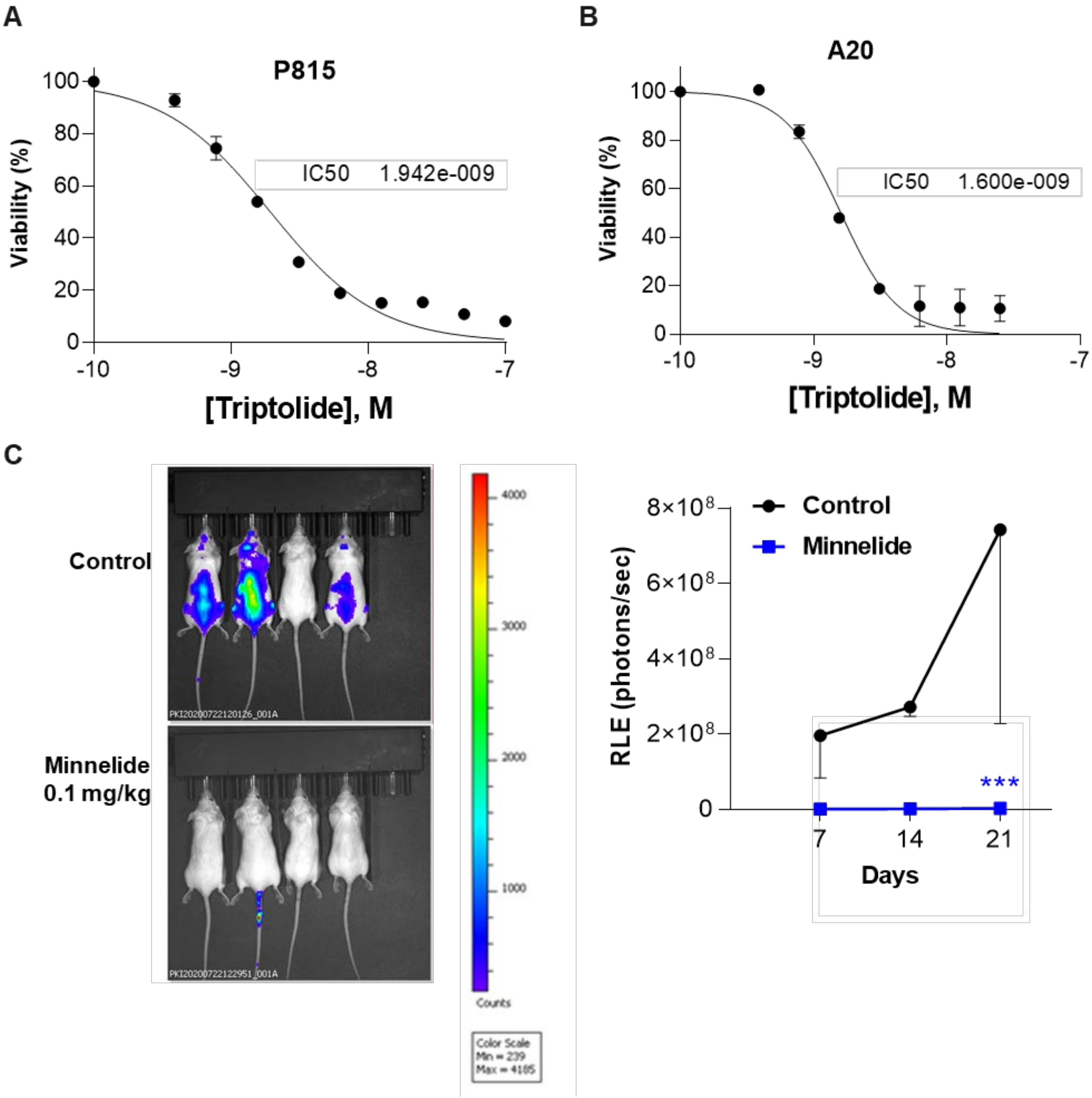


**Figure S7.** ***Anti-tumor activity of Minnelide treatment against experimental cancer cell lines.*** Cytotoxicity assay *in vitro* of (**A**) P-815 mastocytoma and (**B**) A20 B-cell lymphoma (2,000 cells/well) demonstrates triptolide kills both cell lines at comparable IC50 ($1.942x10^{-9}$ vs $1.6\ x10^{-9}$, respectively) at 48 h. (**C**) A20-luciferase expressing tumor cells (2,000) were injected into BALB/c mice and luciferin was administered on Day 7. Minnelide (0.1mg/kg) treatment daily resulted in almost complete absence of detectable A20 cells (n=4 mice/group). Groups were compared using two-tailed unpaired t test. ***p<0.001.

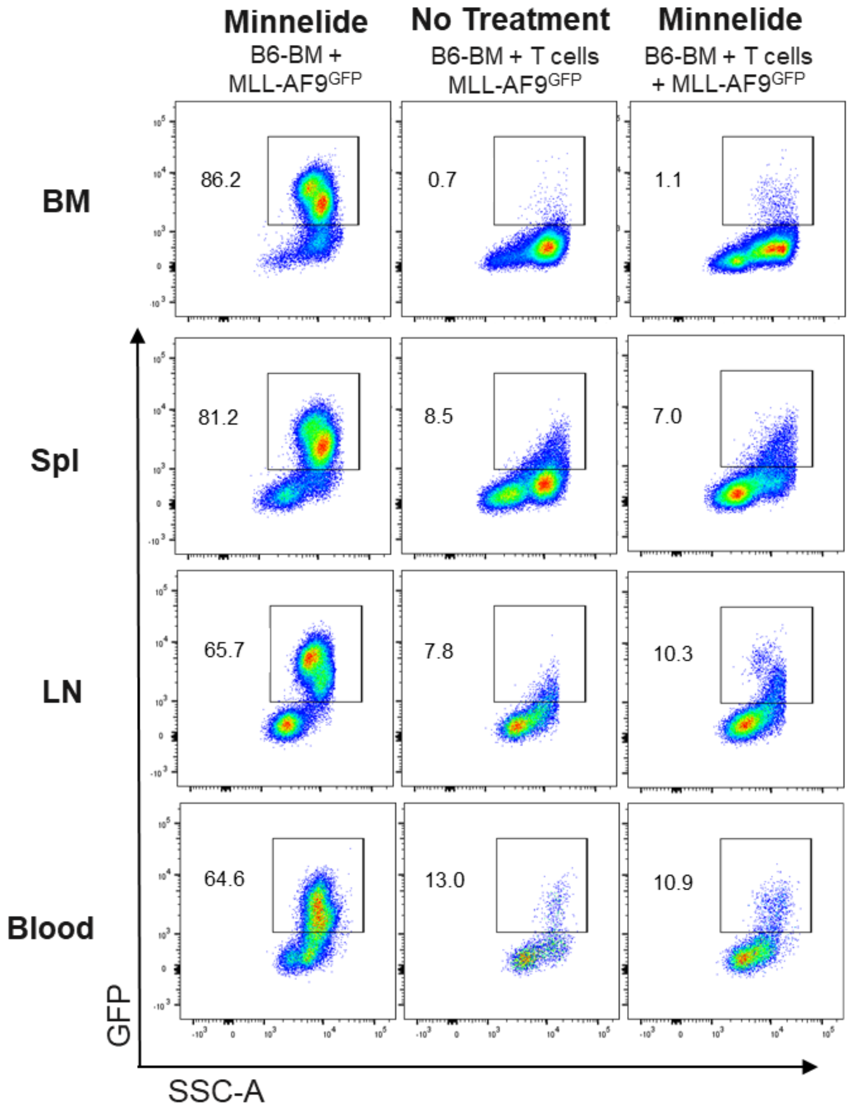


**Figure S8.** ***Minnelide treatment of transplant recipients does not diminish GVL against MLL-AF9 tumor cells.*** Tissues were assessed 40 days post-B6 T + BM cells → BALB/c + MLL-AF9$^{GFP}$ injected recipients. Representative density plot in untreated vs Minnelide-treated mice (0.1mg/kg) revealed comparable frequency of MLL-AF9$^{GFP}$ cells in the BM, spleen, lymph nodes and peripheral blood.

**A Table of % of donor cells**

| | Healthy donor – mobilized |
|---|---|
| WBC (x $10^6$/ml) | 322.5 |
| PBMC (x $10^6$ /ml) | 86 |
| $CD34^+$ cells (x $10^6$ ) | 39.7 |
| % Neutrophils (plus Immature Granulocytes) | 37.6 |
| % Lymphocytes | 34.1 |
| % Monocytes | 28.3 |

**B**

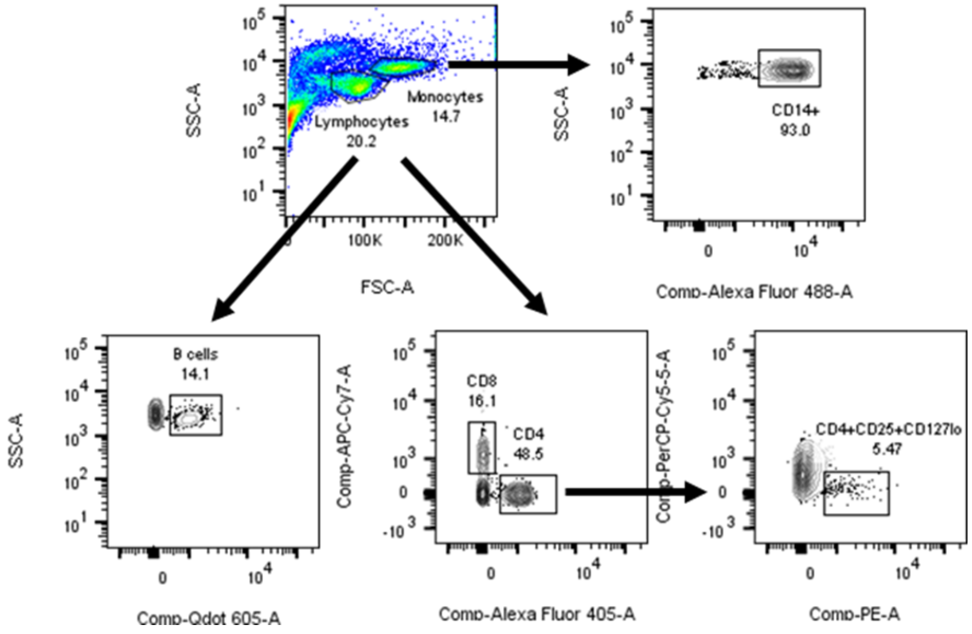


**Figure S9.** ***Analysis of mobilized human peripheral blood used for xenogeneic hematopoietic cell transplants.*** Cryopreserved mobilized peripheral blood sample from a healthy human was obtained from the UM stem cell bank. The sample was thawed and analyzed for immune cell populations. (**A**) Table indicating the percentages of selective cell populations contained in the mobilized peripheral blood sample used for the transplant in “Figure 7”. (**B**) Frequency of CD14+, CD4+, CD8+ and CD4+ Tregs ($CD4^+CD25^+CD127^{lo}$) in the whole blood of the healthy mobilized donor.

**Supplemental Table 1**

| Ab Target / molecule | Fluorophore | Clone | Catalog # | Vendor |
|---|---|---|---|---|
| **Mouse** | | | | |
| CD3 | FITC | 17A2 | 100204 | Biolegend |
| CD4 | BUV805 | GK1.5 | 564922 | BD Biosciences |
| CD4 | FITC | RM4-5 | 100510 | Biolegend |
| CD4 | PacBlue | GK1.5 | 100428 | Biolegend |
| CD4 | PE-Cy7 | RM4-4 | 100528 | Biolegend |
| CD4 | PE-Fire700 | GK1.5 | 100484 | Biolegend |
| CD8a | BUV395 | 53-6.7 | 563786 | BD Biosciences |
| CD8a | PerCP-Cy5.5 | 53-6.7 | 100734 | Biolegend |
| CD8a | PacBlue | 53-6.7 | 100725 | Biolegend |
| CD8a | V500 | 53-6.7 | 560776 | BD Biosciences |
| CD8a | BV510 | 53-6.7 | 100752 | Biolegend |
| CD11b | BV605 | M1/70 | 101257 | Biolegend |
| CD11c | FITC | N418 | 117306 | Biolegend |
| CD19 | AF700 | 6D5 | 115528 | Biolegend |
| CD19 | BV510 | 6D5 | 115546 | Biolegend |
| CD25 | PE-Cy7 | PC61.5 | 25-0251-82 | eBioscience |
| CD25 | APC | PC61 | 557192 | BD Biosciences |
| CD27 | PE-Cy7 | LG.3A10 | 124216 | Biolegend |
| CD44 | APC-Cy7 | IM7 | 103027 | Biolegend |
| CD45.1 | APC | A20 | 110714 | Biolegend |
| CD62L | BV605 | MEL-14 | 104438 | Biolegend |
| CD62L | PECy5 | MEL-14 | 104410 | Biolegend |
| CD90.2 | PacBlue | 53-2.1 | 140306 | Biolegend |
| CD122 | PE | TM-B1 | 553362 | BD Biosciences |
| FoxP3 | eF450 | FJK-16s | 48-5773-82 | Invitrogen |
| FoxP3 | AF700 | MF-14 | 126422 | Biolegend |
| Gata3 | BV421 | 16E10A23 | 653814 | Biolegend |
| Gata3 | PE | 16E10A23 | 653804 | Biolegend |
| $H2K^b$ | FITC | AF6-88.5 | 116506 | Biolegend |
| $H2K^d$ | PE-Cy7 | SF1-1.1 | 116622 | Biolegend |
| ICOS | PerCp e710 | 7E.17G9 | 46-9942-82 | Invitrogen |
| IFN-γ | PE-Cy7 | XMG1.2 | 505826 | Biolegend |
| IFN-γ | BV711 | XMG1.2 | 505836 | Biolegend |

| | | | | |
|---|---|---|---|---|
| IL-13 | PE | JES10-5A2 | 501903 | Biolegend |
| KLRG1 | PE-Cy7 | 2F1 | 138416 | Biolegend |
| Ly6C | PE Dazzle | HK1.4 | 128044 | Biolegend |
| Ly6G | AF700 | 1A8 | 127622 | Biolegend |
| Ly9.1 (CD229.1) | Biotinylated | 30C7 | 557363 | BD Biosciences |
| NK1.1 | PE | PK136 | 108708 | Biolegend |
| NK1.1 | APC | PK136 | 108710 | Biolegend |
| NKp46 | BV421 | 29A1.4 | 137612 | Biolegend |
| RORg | AF647 | Q31-378 | 562683 | BD Biosciences |
| Streptavidin | PE | N/A | 349023 | BD Biosciences |
| Tbet | BV711 | 4B10 | 644820 | Biolegend |
| Viability Dye | UV446 | N/A | L23105 | Invitrogen |
| **Human** | | | | |
| CD4 | BV421 | OKT4 | 317433 | Biolegend |
| CD8 | APC-Cy7 | RPA-T8 | 301016 | Biolegend |
| CD14 | AF488 | M5E2 | 301811 | Biolegend |
| CD19 | BV605 | HIB19 | 302244 | Biolegend |
| CD25 | PECy-7 | BC96 | 302612 | Biolegend |
| CD34 | FITC | 561 | 343604 | Biolegend |
| CD127 | PerCP-Cy5.5 | A019D5 | 986006 | Biolegend |
| | | | | |
